# Exploration of genes encoding KEGG pathway enzymes in rhizospheric microbiome of the wild plant *Abutilon fruticosum*

**DOI:** 10.1186/s13568-024-01678-4

**Published:** 2024-02-21

**Authors:** Aala A. Abulfaraj, Ashwag Y. Shami, Nahaa M. Alotaibi, Maryam M. Alomran, Abeer S. Aloufi, Abeer Al-Andal, Nawwaf R. AlHamdan, Fatimah M. Alshehrei, Fatmah O. Sefrji, Khloud H. Alsaadi, Haneen W. Abuauf, Sahar A. Alshareef, Rewaa S. Jalal

**Affiliations:** 1 Biological Sciences Department, College of Science & Arts, King Abdulaziz University, Rabigh 21911, Saudi Arabia; 2 Department of Biology, College of Science, Princess Nourah bint Abdulrahman University, P.O. Box 84428, Riyadh 11671, Saudi Arabia; 3 Department of Biology, College of Science, King Khalid University, Abha 61413, Saudi Arabia; 4 Dar AlThikr Schools, Jeddah 23526, Saudi Arabia; 5 Department of Biology, Jumum College University, Umm Al-Qura University, P.O. Box 7388, Makkah 21955, Saudi Arabia; 6 Department of Biology, College of Science, Taibah University, Al-Madinah Al-Munawarah 30002, Saudi Arabia; 7 Department of Biological Science, College of Science, University of Jeddah, Jeddah 21493, Saudi Arabia; 8 Department of Biology, Faculty of Applied Science, Umm Al-Qura University, Makkah 24381, Saudi Arabia; 9 Department of Biological Science, College of Science and Arts at Khulis, University of Jeddah, Jeddah 21921, Saudi Arabia

**Keywords:** mWGS, Rhizobiome, Exudation, Biotic stress, Abiotic stress, ABA, ATP

## Abstract

The operative mechanisms and advantageous synergies existing between the rhizobiome and the wild plant species *Abutilon fruticosum* were studied. Within the purview of this scientific study, the reservoir of genes in the rhizobiome, encoding the most highly enriched enzymes, was dominantly constituted by members of phylum Thaumarchaeota within the archaeal kingdom, phylum Proteobacteria within the bacterial kingdom, and the phylum Streptophyta within the eukaryotic kingdom. The ensemble of enzymes encoded through plant exudation exhibited affiliations with 15 crosstalking KEGG (Kyoto Encyclopaedia of Genes and Genomes) pathways. The ultimate goal underlying root exudation, as surmised from the present investigation, was the biosynthesis of saccharides, amino acids, and nucleic acids, which are imperative for the sustenance, propagation, or reproduction of microbial consortia. The symbiotic companionship existing between the wild plant and its associated rhizobiome amplifies the resilience of the microbial community against adverse abiotic stresses, achieved through the orchestration of ABA (abscisic acid) signaling and its cascading downstream effects. Emergent from the process of exudation are pivotal bioactive compounds including ATP, D-ribose, pyruvate, glucose, glutamine, and thiamine diphosphate. In conclusion, we hypothesize that future efforts to enhance the growth and productivity of commercially important crop plants under both favorable and unfavorable environmental conditions may focus on manipulating plant rhizobiomes.

**Supplementary Information:**

The online version contains supplementary material available at 10.1186/s13568-024-01678-4.

## Introduction

The wild plant species *Abutilon fruticosum*, distinguished by its robust xerophytic attributes, holds a position as a perennial herbaceous specimen within the esteemed family Malvaceae. This botanical entity has garnered substantial attention, attributed to its multifaceted medicinal and economic properties, as previously documented (Patel and Rajput 2013). Endemic to the designated realms of southwestern and western Asia, as recently outlined (Alzahrani 2021), the plant boasts a remarkable array of invaluable steroids, carbohydrates, flavonoids, linoleic acid, oleic acid, and palmitic acid, residing prominently within its leaves and seeds (Suryawanshi and Umate 2020). The leaf extract derived from *A. fruticosum* has secured notable accolades for its potent antimicrobial efficacy against a spectrum of respiratory tract infections caused by *Escherichia coli*, *Pseudomonas aeruginosa*, *Streptococcus epidermidis*, and *Candida tropicalis* (Gouda et al. 2022). Furthermore, *A. fruticosum*, marked by its intrinsic lack of toxicity, is ingrained within the tapestry of traditional and folk medicine, serving as a venerable therapeutic measure for afflictions such as bodily pain, ulcers, hemorrhoids, and inflammation of the human bladder (Patel and Rajput 2013).

The advent of next-generation sequencing methodologies, encompassing the revered domain of 16S rRNA amplicon sequencing and metagenomic whole genome shotgun sequencing (mWGS), has inaugurated a new epoch of profound understanding concerning the configuration and functionality of the microbial consortium enveloped within the rhizospheric soil. The establishment of comprehensive and informative databases, such as Greengenes previously curated (DeSantis et al. 2006), and the SILVA database previously assembled (Carlton et al. 2002), incorporating a vast repertoire of marker genes hailing from diverse taxa, bears testament to the great advantages engendered by the 16S rRNA methodology. However, the inherent constraint of the 16S rRNA approach lies in its incapability to discern viral entities due to their absence of conserved genes analogous to the 16S or 18S rRNA genes, which serve as universal markers across diverse taxonomic ranks. Discriminating research undertaken by a cohort of intrepid investigators (Chen et al. 2021; Claesson et al. 2010; Kennedy et al. 2014) has unequivocally demonstrated the comparative insensitivity and paucity of substantive functional insights originating from the 16S sequencing modality. Conversely, the deployment of mWGS has surfaced as an effective panacea, conferring a heightened level of precision and enabling the delineation of draft microbial genomes. Furthermore, the mWGS concurrently provides a profusion of insights into the authentic gene abundance and metabolic cascades permeating the resident microbiomes within a given ecological niche. This pivotal truth has been recently substantiated by the pioneering efforts of several investigators (Dilthey et al. 2019, Quince et al. 2017, Raes et al. 2007, Scholz et al. 2016, Stewart et al. 2018, Tringe et al. 2005, Wilkins et al. 2019).

Conventional agricultural practices, often employed to augment crop yield, wield a substantial impact upon the soil microbiome associated with cultivated plant species, frequently leading to the inadvertent depletion of crucial microorganisms pivotal for enhancing plant growth and safeguarding against virulent pathogens (Hartman et al. 2017, Kolton et al. 2012; Pérez-Jaramillo et al. 2017; Yin et al. 2013). In contrast, the rhizobiome exhibited by wild plant species, exemplified poignantly by *A. fruticosum*, remains impeccably preserved within its natural habitat, characterized by an unadulterated assembly concerning structural intricacies, microbial community assembly patterns, molecular dynamics, plant–microbe interactions, and the dynamic evolutionary trajectories of the resident microbial consortia (Bulgarelli et al. 2015, Pett-Ridge and Firestone 2017, Pett-Ridge et al. 2021, Schlaeppi et al. 2014, Zachow et al. 2014). Consequently, the microbiomes harbored by wild plant species stand as a fertile reservoir, housing a plethora of beneficial microorganisms and offering an unparalleled opportunity for the discovery of novel antibiotics.

The present study aimed at unraveling the intricate tapestry of metabolites and metabolic processes pervading the interplay between the wild plant species *A. fruticosum* and the intact microbial assemblage within its rhizospheric microcosm. We believe that the relationship between the plant and the rhizobiome in its surroundings can be advantageous to both parties.

## Materials and methods

### Sample collection and DNA extraction

The acquisition of samples was undertaken in triplicates from the rhizosphere soil milieu of individual *Abutilon fruticosum* plants, flourishing naturally within the North Western Mecca province of Saudi Arabia. These samples were situated in close proximity to the coastal precincts of the Red Sea, as meticulously documented (Al-Eisawi and Al-Ruzayza 2015). The chosen collection locales for the distinct soil typologies had encountered an extended period of precipitation deficiency spanning over three months preceding sample collection. In the case of rhizosphere soil, the lateral roots were excised at a depth ranging from 10 to 30 cm. Subsequently, soil encompassing a radius of ≤ 1 cm from the root periphery, albeit physically distinct from the root itself, was painstakingly procured. In contrast, bulk soil samples were obtained at a minimum separation of 10 m from *A. fruticosum* plants. The collected soil samples were expeditiously immersed in liquid nitrogen, transported to the laboratory, and conserved at a temperature of − 20 °C until the commencement of DNA extraction, following the procedural guidelines as described (Hurt et al. 2001). The DNA extraction buffer composition comprised 10 ml Tris–HCl (1 M), 4 ml M EDTA (0.5), 2 g CTAB, and 28 ml NaCl (5 M). Following centrifugation at 12,000 ×*g*, the extracted DNAs were precipitated, washed with 0.5 ml chilled ethanol (70%), and resuspended in 1 ml TE buffer. Prior to incubation at 37 °C, the DNAs were subjected to RNase A treatment at 10 um concentration to eliminate any residual RNA. Subsequent to this, electrophoresis on a 1% agarose gel was conducted for validation of the integrity and purity of the isolated DNAs. Each DNA sample was adjusted to a concentration of 10 ng/μl utilizing the dsDNA Assay kit (Life Technologies, Carlsbad, CA, USA) according to manufacturer's stipulations, in preparation for whole metagenome shotgun sequencing (mWGS) as elucidated (Doyle and Doyle 1987). Thereafter, 30 μl of each DNA extract was dispatched to Novogene Co. (Singapore) for subsequent deep sequencing.

### Deep sequencing and bioinformatics analysis

The DNA entities underwent sequencing on the Illumina HiSeq 2500 platform subsequent to the formulation of libraries employing the Ultra DNA Library Prep kit for Illumina (NEB, Ipswich, MA, USA). The resultant raw sequencing data were duly archived in the European Nucleotide Archive (ENA) with accession numbers ERS15580318-23, respectively for rhizosphere soil samples R1, R2, and R3, as well as bulk soil samples S1, S2, and S3. Sequences with low-quality bases characterized by Q values ≤ 38, surpassing the 40-bp threshold were excised and expunged post physical segregation of the DNAs. Additionally, reads containing ≥ 10 bp of undetermined bases (Ns) were removed. The high-quality reads were then subjected to de novo assembly utilizing MEGAHIT with a K-mer size of 55 to recuperate scaffolds, whereas removal of chimeric sequences was undertaken in consonance with outlined methodologies (Karlsson et al. 2012; Mende et al. 2012; Oh et al. 2014). The less abundant, unassembled reads from all samples were amalgamated and subjected to de novo re-assembly to engender NOVO_MIX scaffolds. These assemblages were subsequently truncated at 'N'-containing regions to yield fragments referred to as scaftigs, a concept previously expounded (Mende et al. 2012; Nielsen et al. 2014). NOVO_MIX scaftigs that were present in two or more samples were further analyzed, and the cleansed data was next subjected to mapping using Soap 2.21. Predictive annotation of genes for the generated scaftigs was executed utilizing MetaGeneMark, as elaborated (Nielsen et al. 2014). The ensuing gene set was subjected to dereplication via the Cluster Database at High Identity with Tolerance (CD-HIT) methodology as described (Fu et al. 2012; Li and Godzik 2006). Thereafter, gene redundancy was ameliorated via a greedy pairwise comparison, culminating in the formation of non-redundant gene catalogs (nrGC), in consonance with previous methodologies (Li et al. 2014).

### Gene annotation and functional interpretation

The gene annotation endeavor was realized through the MEGAN binning reference-based classification method, as previously delineated (Huson et al. 2016; Huson et al. 2011). Subsequently, functional analyses of coding metagenomic sequences culled from microbiome samples were conducted and predicated on their semblance to sequences resident within the Kyoto Encyclopedia of Genes and Genomes (KEGG) databases. Particular emphasis was vested in the core databases, namely KEGG PATHWAY, KEGG orthology (KO), and KEGG ENZYME (EC). These databases served to infer the functions of coding metagenomic sequences from diverse microbiome samples, as previously expounded (Kanehisa et al. 2006, 2014; Kanehisa et al. 2016a, 2016b; Segata et al. 2011). The KO database aided in identifying molecular functions epitomized by functional orthologs, while the KEGG PATHWAY database facilitated the mapping of pathways at three discrete hierarchical tiers (levels 1, 2, and 3). The KEGG ENZYME (EC) database was instrumental in the functional annotation, as previously detailed (Karlsson et al. 2012; Karlsson et al. 2013; Li et al. 2014). The cumulative utilization of these three databases conferred an all-encompassing profile elucidating the functions of the two soil microbiome categories at KEGG levels 1, 2, and 3, as well as at the EC level. Subsequently, a cluster analysis predicated on function abundance data was conducted employing the Bray–Curtis distance metric, then principal component analysis (PCA) was performed. Ultimately, heatmaps were generated to illustrate the distribution of highly abundant functions across the three KEGG levels (KEGG category or level 1, KEGG sub-category or level 2, and KEGG pathway or level 3), in addition to the EC level for KEGG enzymes.

## Results

### Fidelity of raw sequences datasets

**Fig. 1:**
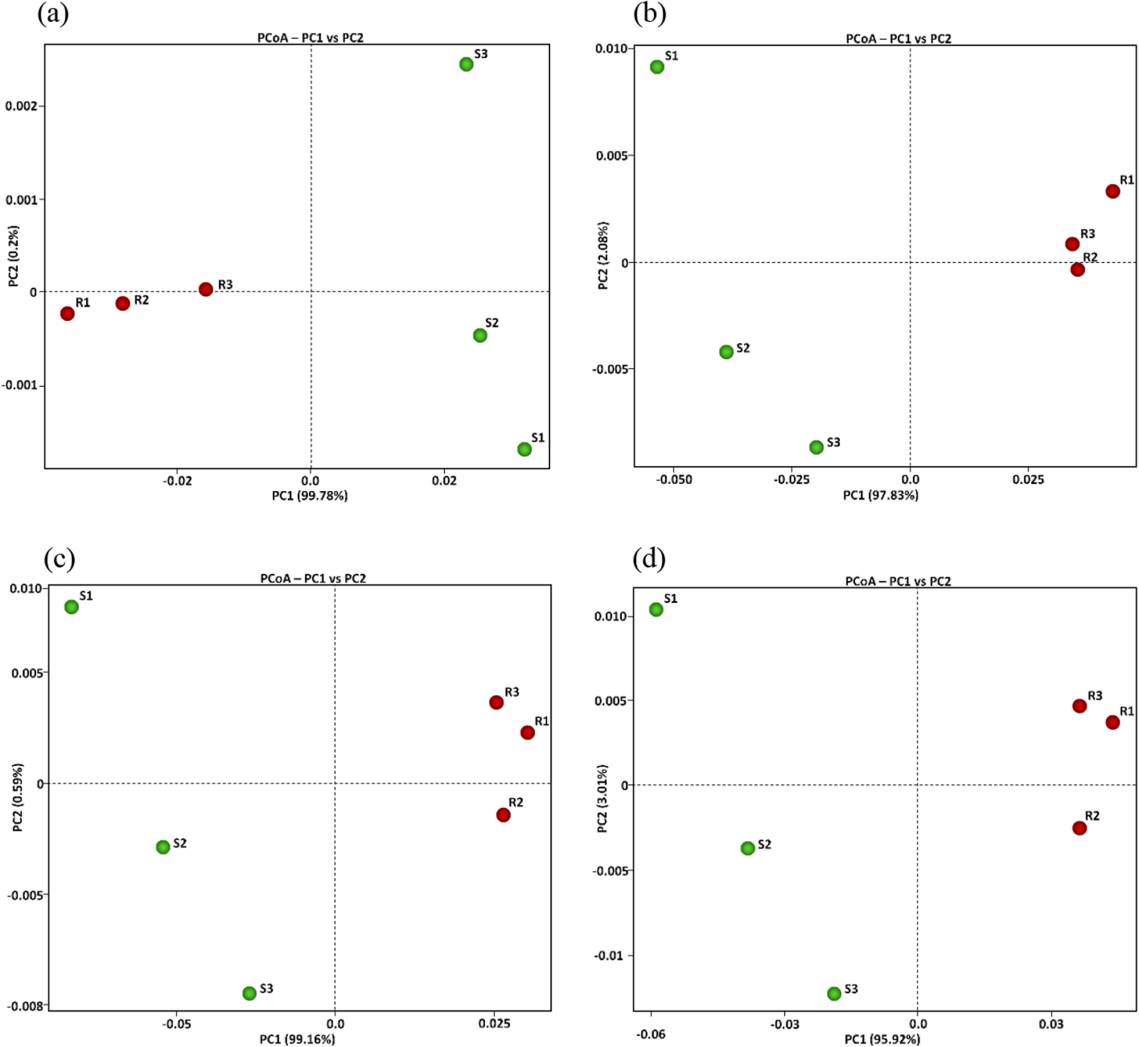
Principal coordinate analysis (PCoA) based on KEGG (Kyoto Encyclopedia of Genes and Genomes) database Levels 1 (**a**), 2 (**b**), 3 (**c**), and EC (d) pertaining to microbiome samples of rhizospheric (R) and bulk (S) soils of *A. fruticosum*

To ensure the fidelity and accuracy of the KEGG enzyme raw datasets underpinning this inquiry, a meticulous assessment using principal component analysis (PCA) was executed. The purpose was to unravel the alignment amidst samples concerning the microbiome structure and the prevalence of genes within each soil type, more specifically in the stratified tiers of KEGG Levels (1, 2 and 3) and EC (Fig. 1).

This scrutiny unmistakably brought to light a stark differentiation between the microbiomes intrinsic to the two divergent soil types. Evidently, the microbiome samples from the rhizosphere conspicuously clustered within the positive quadrant of the PCA 1 (PC1) concerning KEGG Levels 2, 3, and EC (Fig. 1a). Simultaneously, these samples were situated within the negative side for KEGG Level 1. In vivid contrast, the bulk soil microbiome samples showcased an opposing trend across all four KEGG levels (Fig. 1a–d). Noteworthy is the high diversity in terms of PC2 observed in the bulk soil microbiome as opposed to the rhizosphere soil microbiome.

### Statistics of assembled raw sequences datasets

The assemblage of open reading frames (ORFs), representing the query sequences, were meticulously aligned against analogous sequences available within the National Center for Biotechnology Information (NCBI) database, constituting the subject sequences. This alignment provided intricate insights into diverse parameters encompassing identity/mismatch, gap sizes quantified in nucleotides (nt), as well as the precise genomic coordinates demarcating the gene's initiation and termination sites (Additional file 2: Table S1). The amassed dataset encompassed a total of 270,863 ORFs, emanating from a sole sample sourced from either of the soil microbiomes, whereas 777,711 ORFs were attributed to two or more samples arising from either or both soil microbiome types (e.g., NOVO_MIX). Throughout the alignment, query sequences exhibited sizes spanning from 27 to 4445 nt, with identity gradients spanning from 50 to 100%, and mismatch magnitudes oscillating from 0 to 1286 nt. A salient point to underscore is the fact that a given subject entity might yield outcomes from multiple queries. A conspicuous instance of this scenario is manifested in the subject aaa:Acav_0015, demonstrating matches for two queries, namely NOVO_MIX_1208907 and NOVO_MIX_636028 (Additional file 2: Table S1).

### Detection and abndance of genes encoding KEGG pathway enzymes

**Fig. 2:**
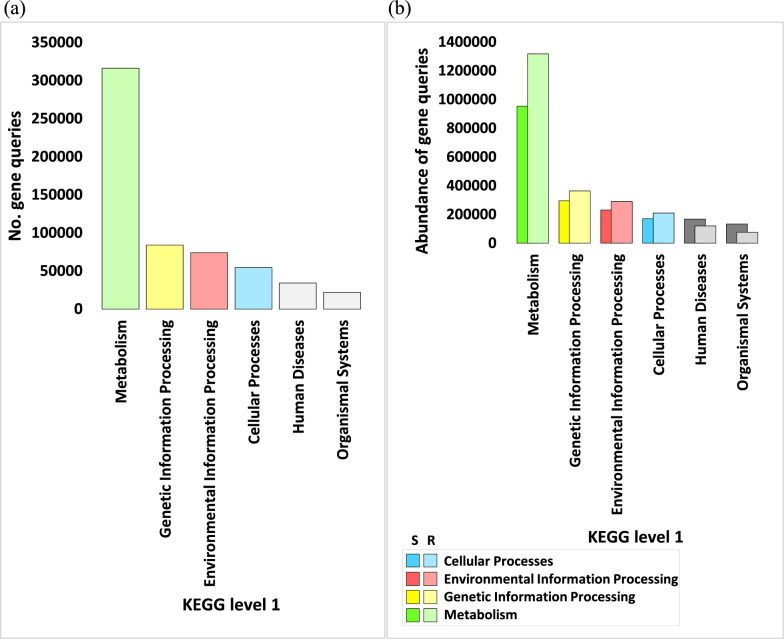
Number (**a**) and Abundance (**b**) of genes encoding enzymes of diverse functional categories of KEGG Level 1 in microbiome samples derived from rhizospheric (R) and adjacent bulk soil (S) environments of *A. fruticosum*. Gray-shaded columns constitute less abundant KO categories ( < 50,000). Elaborated insights pertaining to the number and abundance of genes are accessible in Additional file 2: Tables S2 and S3, Respectively

**Fig. 3:**
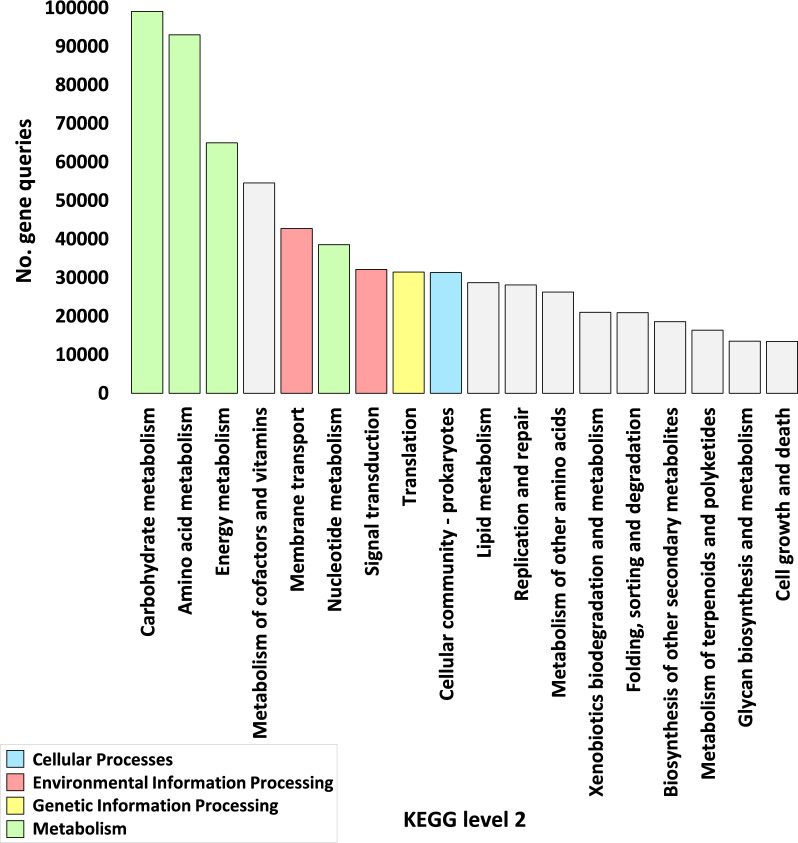
Number of genes encoding enzymes in highly abundant functional sub-categories of KEGG Level 2 (> 10,000) across microbiome samples of rhizospheric (R) and surrounding bulk soil (S) of *A. fruticosum*. Gray-shaded columns constitute either less abundant KO sub-categories or those housing less abundant enzymes. Additional elucidation is provided in Additional file 2: Tables S4 and S5

**Fig. 4:**
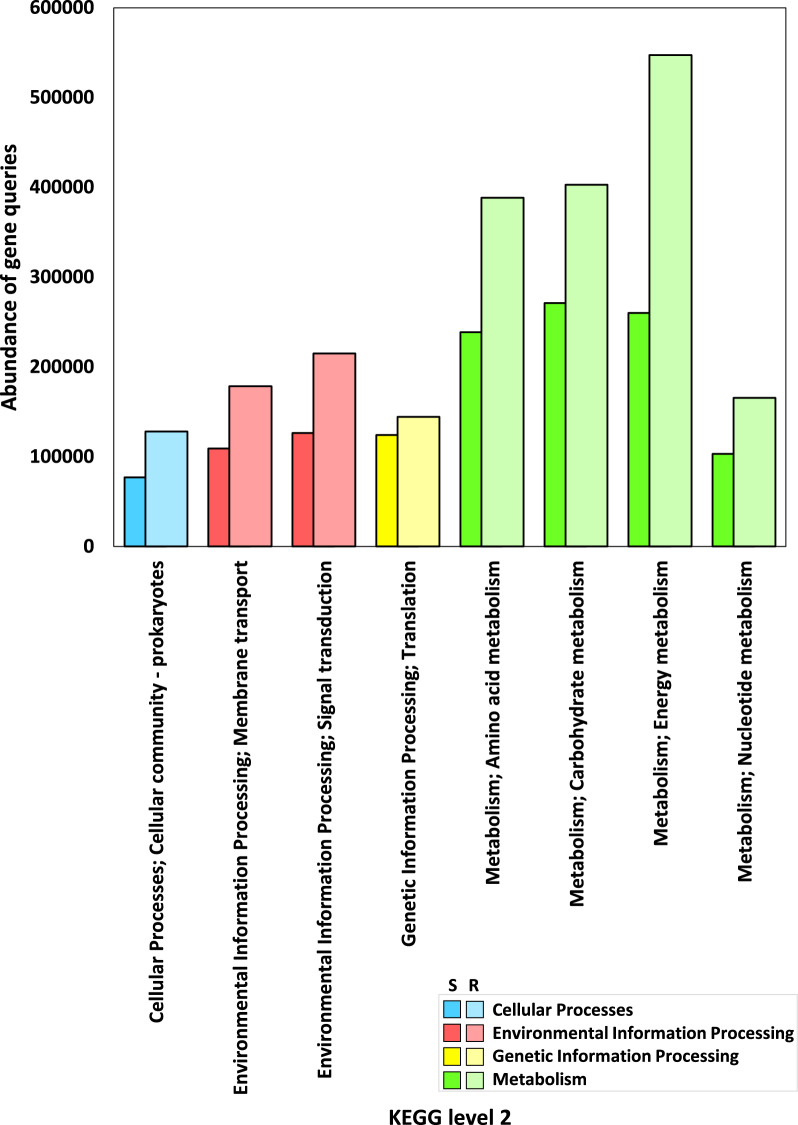
Abundance of genes encoding enzymes within highly abundant functional sub-categories of KEGG Level 2 across microbiome samples of rhizospheric (R) and adjacent bulk soil (S) environments of *A. fruticosum*. Further discernment is accessible through Additional file 2: Tables S6 and S7

**Fig. 5:**
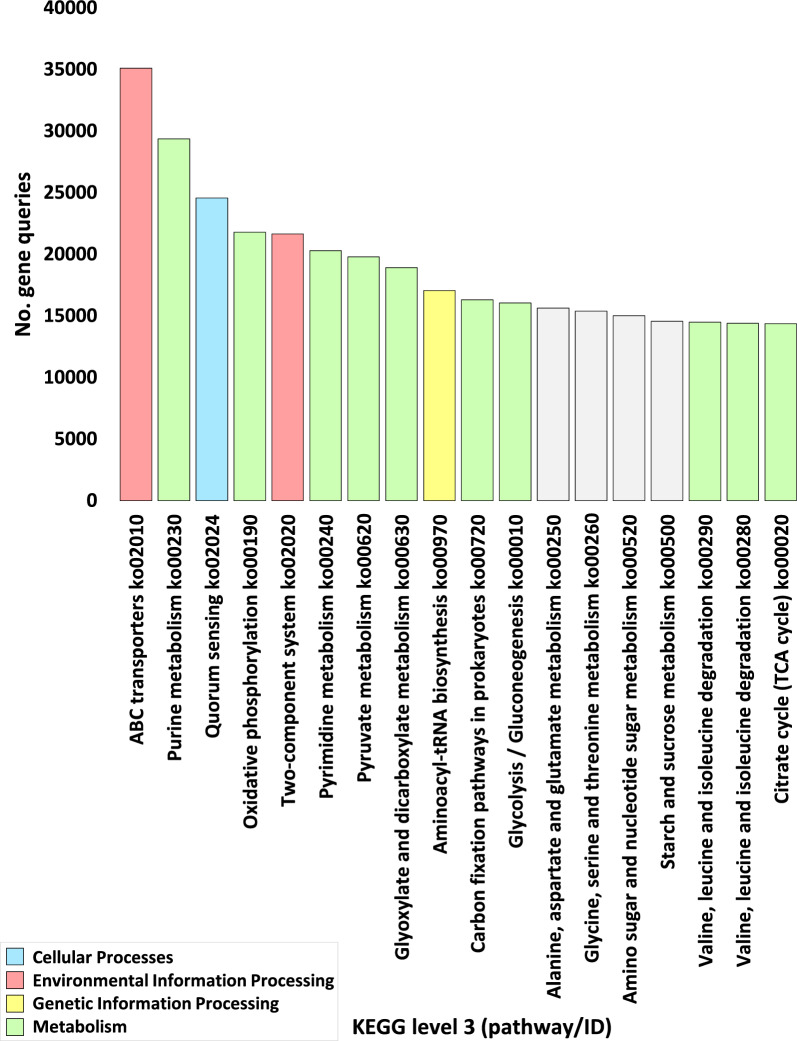
Number of genes encoding enzymes within highly abundant KEGG pathways (> 14,000) in microbiome samples derived from rhizospheric (R) and surrounding bulk soil (S) of *A. fruticosum*. Grey-shaded color designates pathways housing less abundant enzymes. Elaborative Information is present in Additional file 2: Tables S8 and S9

**Fig. 6:**
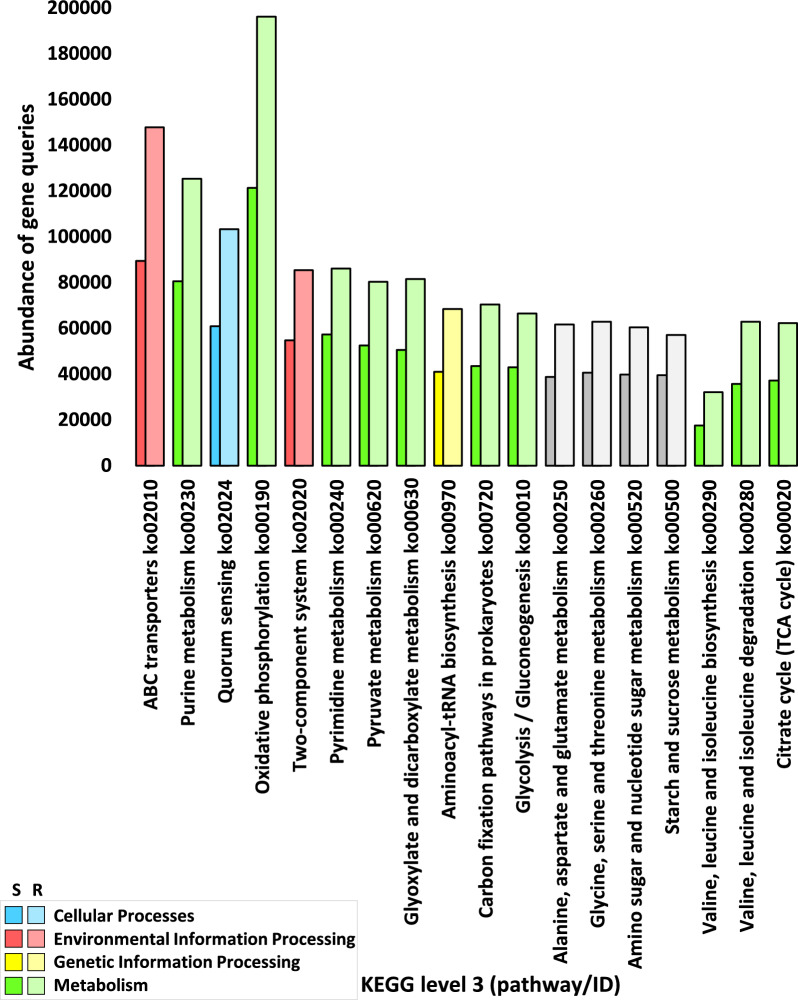
Abundance of genes encoding enzymes within highly abundant KEGG pathways between microbiome samples of rhizospheric (R) and bulk (S) soils of *A. fruticosum*. Additional file 2: Tables S10 and S11 offer further insights. Shades of light (rhizosphere) and dark (bulk) gray indicate differential abundance of pathways with less abundant enzymes

**Fig. 7:**
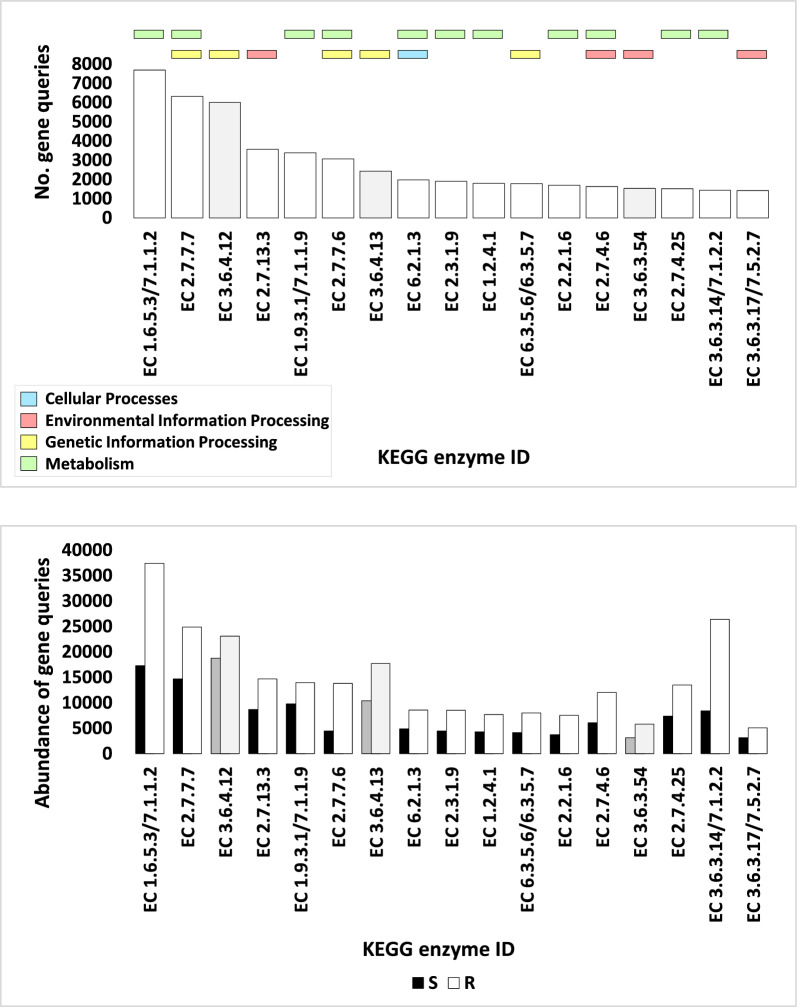
Number (**a**) and abundance (**b**) of genes encoding highly abundant enzymes (> 1400) across interconnected KEGG pathways in microbiome samples of rhizospheric (R) and adjacent bulk soil (S) environments of *A. fruticosum*. Whitened and blackened shades denote enzymes present in highly abundant KEGG pathways (> 14,000), while light and dark gray shades symbolize enzymes existing within less abundant KEGG pathways. Enzyme abundance is enumerated in Additional file 2: Table S15. EC 1.6.5.3/7.1.1.2 = NADH-quinone oxidoreductase subunit J, EC 2.7.7.7 = DNA polymerase III subunit delta, EC 3.6.4.12 = ATP-dependent DNA helicase RecG, EC 2.7.13.3 = two-component system, OmpR family, phosphate regulon sensor histidine kinase PhoR, EC 1.9.3.1/7.1.1.9 = cytochrome c oxidase subunit IV, EC 2.7.7.6 = DNA-directed RNA polymerase subunit beta', EC 3.6.4.13 = pre-mRNA-splicing factor ATP-dependent RNA helicase DHX16; ATP-dependent RNA helicase RhlE, EC 6.2.1.3 = long-chain acyl-CoA synthetase, EC 2.3.1.9 = acetyl-CoA C-acetyltransferase, EC 1.2.4.1 = pyruvate dehydrogenase E1 component beta subunit, EC 6.3.5.6/6.3.5.7 = aspartyl-tRNA(Asn)/glutamyl-tRNA(Gln) amidotransferase subunit B, EC 2.2.1.6 = acetolactate synthase I/II/III large subunit, EC 2.7.4.6 = nucleoside-diphosphate kinase, EC 3.6.3.54 = Cu + -exporting ATPase, EC 2.7.4.25 = CMP/dCMP kinase, EC 3.6.3.14/7.1.2.2 = F-type H + -transporting ATPase subunit beta, EC 3.6.3.17/7.5.2.7 = ribose transport system ATP-binding protein. Names of all the enzymes are shown in Additional file 2: Table S15. Further details concerning gene number are detailed in Additional file 2: Table S12, whereas abundance information is presented in Additional file 2: Table S14

The number of genes pertaining to KEGG enzymes at levels 1, 2, 3, and EC, within categories, sub-categories, pathways, and enzymes respectively, across assorted soil microbiome samples, is vividly illustrated in Figs. 2a, 3, 5, and 7a, with further detailed exposition provided in Tables S2, S4, S8, and S12 respectively. While, abundance of genes pertaining to KEGG enzymes at levels 1, 2, 3, and EC, within categories, sub-categories, pathways, and enzymes respectively, across assorted soil microbiome samples, is illustrated in Figs. 2b, 4, 6, and 7b, with further detailed exposition provided in Tables S3, S5, S11, and S14, respectively.

An all-encompassing compilation of gene statistics for the entire set of enzyme ECs is meticulously presented in Additional file 2: Table S13. Additional file 1: Fig. S1 delineates the gene query data at levels 1 and 2. The conspicuous outcomes, as evident from Figs. 2 and Additional file 2: Table S2, underscore a discernible superfluity of genes, exceeding 50,000, residing within the KEGG category delineated as ‘Metabolism,’ followed in sequence by categories tagged as ‘Genetic information processing,’ ‘Environmental information processing,’ and ‘Cellular processes.’ The highly enriched sub-categories, surpassing 10,000 instances, were further selected for in-depth examination (Fig. 3 and Additional file 2: Table S5). These preferred sub-categories, representing elevated enrichment levels within the category ‘Metabolism,’ encompass ‘Carbohydrate metabolism,’ ‘Amino acid metabolism,’ ‘Energy metabolism,’ and ‘Nucleotide metabolism.’

Additionally, the sub-category ‘Translation’ emerges within the ‘Genetic information processing’ category, while the KEGG sub-categories ‘Membrane transport’ and ‘Signal transduction’ are ensconced within the ‘Environmental information processing’ category. Further, the sub-category ‘Cellular community-prokaryotes’ is found within the ‘Cellular Processes’ category (Fig. 3 and Additional file 2: Table S5). Among the 18 highly prevalent KEGG pathways (exceeding 14,000 occurrences), 14 are earmarked for extended exploration (Fig. 5 and Additional file 2: Tables S8 and S9) where four pathways fail to exhibit any instances of highly enriched enzymes (Fig. 5 and Additional file 2: Table S9). Among these 14 KEGG pathways, 10 reside within the 'Metabolism’ category, while one pathway is nestled within the ‘Genetic information processing’ category, two pathways abide in the ‘Environmental information processing’ category, and one pathway aligns with the ‘Cellular processes’ category (Fig. 5 and Additional file 2: Table S9). Remarkably, the scrutiny of KEGG enzymes underscores substantial enrichment (surpassing 1400 occurrences) in the case of 17 enzymes (Fig. 7a and Additional file 2: Table S12).

Pathways of three enzymes demonstrate subdued enrichment levels (below 14,000 occurrences). These three enzymes are consequently excluded from further exploration (Fig. 7a and Additional file 2: Table S16). The 'Metabolism' category encompasses seven of the remaining 14 enzymes (e.g., IDs EC 1.6.5.3/7.1.1.2, EC 1.9.3.1/7.1.1.9, EC 2.3.1.9, EC 1.2.4.1, EC 2.2.1.6, EC 2.7.4.25, and EC 3.6.3.14/7.1.2.2), while the 'Genetic information processing' category features one enzyme (e.g., ID EC 6.3.5.6/6.3.5.7), and the 'Environmental information processing' category comprises two enzymes (e.g., IDs EC 2.7.13.3 and EC3.6.3.17/7.5.2.7) (Fig. 7). The remaining four enzymes, identified by the IDs EC 2.7.7.7, 2.7.7.6, 6.2.1.3, and 2.7.4.6, span across the ‘Metabolism’ and ‘Genetic information processing,’ ‘Metabolism’ and ‘Cellular processes,’ and ‘Metabolism’ and ‘Environmental information processing’ categories, respectively (Fig. 7a). For a comprehensive listing of enzymes and their associated ECs residing within the microbiomes of *A. fruticosum*, participating in one or more categories, Additional file 2: Table S15 serves as an indispensable reference.

Thorough evaluation of comparative gene abundance between rhizosphere and bulk soil microbiome samples of *A. fruticosum*, spanning four distinct KEGG levels (level 1, 2, 3, and EC) was done. Gene abundance between rhizosphere and bulk soil microbiome samples of *A. fruticosum* for the four KEGG levels 1, 2, 3 and EC is shown, respectively, in Figs. 2b, 4, 6 and 7b and in Additional file 2: Tables S3, S6, S10 and S14.

Selection of KEGG categories (Additional file 2: Table S3), KEGG sub-categories (Additional file 2: Table S7), KEGG pathways (Additional file 2: Table S11) and KEGG enzymes (Additional file 2: Table S16) was based on the results of gene number at the four KEGG levels. Abundance was shown to be higher in the four KEGG categories (Figs. 2b and Additional file 1: Fig. S2), the eight KEGG sub-categories (Figs. 4 and Additional file 1: Fig. S3), the 14 KEGG pathways (Figs. 6 and Additional file 1: Fig. S4) and the 14 KEGG enzymes (Figs. 7b and Additional file 1: Fig. S5). Additional file 1: Figs. S6–S19 display the 14 pathways that present the levels of various enzyme enrichment (referring to gene query number) in the rhizospheric microbiome. The enrichment level is indicated by colored boxes around the enzyme EC (or metabolite); red indicates a higher enrichment level in the rhizospheric soil microbiome of *A. fruticosum* when compared to the bulk soil microbiome, and blue indicates a lower enrichment level when compared to the bulk soil microbiome. While only a few of the enzymes and metabolites displayed extremely high abundance (Fig. 7b), the majority of them displayed high enrichment in the rhizospheric soil microbiome (Additional file 1: Figs. S6–S20). When compared to the bulk soil microbiome, the 14 most highly abundant KEGG enzymes showed higher enrichment levels in the rhizospheric microbiome. We also utilized the less enriched KEGG pathway “Fatty acid biosynthesis” of sub-category “Lipid metabolism” (category “Metabolism”) to investigate the highly enriched enzyme’s function further (Additional file 1: Fig. S20), which revealed a gene count of less than 1400 (Additional file 2: Table S12), e.g., long-chain acyl-CoA synthetase (EC 6.2.1.3) of this pathway.

Additional file 2: Table S17 provides the findings for microbes that produce the highly abundant enzymes in rhizosphere soil microbiome of *A. fruticosum*. It is interesting to note that the most abundant enzyme, e.g., NADH-quinone oxidoreductase subunit J (EC 1.6.5.3/7.1.1.2), is only found in eukaryotic microbes. Another enzyme, called cytochrome c oxidase subunit III (EC 1.9.3.1/7.1.1.9), is also unique to eukaryotic microbes. One enzyme, e.g., nucleoside-diphosphate kinase (EC 2.7.4.6), was found only in bacteria-related microbes. The two kingdoms of Eukaryota and Bacteria both contain the four enzymes long-chain acyl-CoA synthetase (EC 6.2.1.3), pyruvate dehydrogenase E1 component beta subunit (EC 1.2.4.1), F-type H + -transporting ATPase subunit beta (EC 3.6.3.14/7.1.2.2), and ribose transport system ATP-binding protein (EC 3.6.3.17/7.5.2.7). Only one enzyme, e.g., CMP/dCMP kinase (EC 2.7.4.25), was found in the two bacterial and archaeal kingdoms. The remaining six highly abundant enzymes, namely DNA polymerase III subunit delta (EC 2.7.7.7), two-component system phosphate regulon sensor histidine kinase (OmpR family) PhoR (EC 2.7.13.3), DNA-directed RNA polymerase subunit beta' (EC 2.7.7.6), acetyl-CoA C-acetyltransferase (EC 2.3.1.9), aspartyl-tRNA(Asn)/glutamyl-tRNA(Gln) amidotransferase subunit B (EC 6.3.5.6/6.3.5.7) and acetolactate synthase I/II/III large subunit (EC 2.2.1.6), exist in phyla of the three kingdoms. Phyla of kingdom Archaea, commonly contain highly abundant enzymes, include Euryarchaeota, and Thaumarchaeota, while those of kingdom Bacteria include Acidobacteria, Bacteroidetes, Candidatus Saccharibacteria, Chloroflexi, Cyanobacteria, Firmicutes, Gemmatimonadetes, Planctomycetes and Proteobacteria, and those of kingdom Eukaryota include Arthropoda, Ascomycota, Basidiomycota, Chordata, Mucoromycota, Nematoda and Streptophyta. The most abundant of which are phylum Thaumarchaeota of kingdom Archaea, phylum Proteobacteria of kingdom Bacteria and phylum Streptophyta of kingdom Eukaryota (Additional file 2: Table S17).

## Discussion

The current investigation identified several enriched enzymes associated with 15 crosstalking KEGG pathways. Because of plant root exudation, the number and abundance of genes encoding these enzymes vary between the two types of soil. This kind of symbiotic connection seems to be controlled by ABA signaling, which can then triggers further processes that improve the plant's tolerance to harmful abiotic stimuli. The present study indicated that this plant–microbe association can result in the biosynthesis of several important bioactive compounds including glutamine, ATP, D-ribose, pyruvate, glucose, and thiamine diphosphate.

It was previously established that human-mediated agricultural practices have a significant impact on the rhizobiome of cultivated crop plants, often leading to the disruption and alteration of the original natural microbial community that resides in the rhizosphere (Hartman et al. 2017; Kolton et al. 2012; Pérez-Jaramillo et al. 2017; Yin et al. 2013). A meticulous exploration of the microbial gene composition within the rhizosphere of *A. fruticosum*, as compared to bulk soil, is thus undertaken, considering the distinct chemical milieu influenced by plant exudates and root excretion processes (Pett-Ridge et al. 2021).

The rhizosphere, often referred to as the ‘root-soil interface,’ is an ecological hotspot where intricate interactions between plants and a diverse microbial community take place (Berendsen et al. 2012; Sugiyama 2019; Sugiyama et al. 2014). This zone acts as an auxiliary genome, similar in function to the human microbiota, providing a reservoir of advantageous traits that promote growth and survival in a range of environments (Berendsen et al. 2012). Numerous studies have emphasized the crucial influence of plant root architecture and the composition of root exudates on shaping the microbial community in the rhizosphere, playing a fundamental role in attracting beneficial bacteria and providing essential substrates for their growth (Huang et al. 2014; Saleem et al. 2018). Consequently, it is anticipated that there will be distinctive microbial community signatures between different soil types (bulk vs. rhizosphere) and influenced by the genetic makeup of the host plant, which ultimately govern the composition of the rhizospheric microbiome (Holden 2019).

In the context of our study, a comparative analysis between the rhizospheric and bulk soil samples of *A. fruticosum* reveals a notable enrichment of the enzyme long-chain acyl-CoA synthetase (ACSL or RpfB; EC 6.2.1.3) within the ‘Quorum Sensing’ (QS) pathway specifically in the rhizospheric soil (as illustrated in Fig. 7). Remarkably, this enzyme, also known as the regulator of pathogenicity factor B (RpfB) in *Xanthomonas campestris* (Bi et al. 2014), plays a pivotal role in regulating bacterial population density across different ecological niches through complex gene regulation mechanisms (Rutherford and Bassler 2012). The expression of these genes is modulated by small signaling molecules called autoinducers (AIs), which are secreted into the environment mostly by bacteria and function as communication molecules (Rutherford and Bassler 2012).

Plants produce a diverse array of secondary metabolites, some of which have antimicrobial properties that disrupt the ability of cohabiting phytopathogens to achieve quorum, thereby protecting plant cells from potential harm (Rudrappa and Bais 2008). The *rpfB* gene is part of a cluster of genes that includes *rpfF*, *rpfC*, and *rpfG* (Additional file 1: Fig. S6), collectively responsible for the production and detection of diffusible signaling factors (DSFs)—fatty acid signaling molecules crucial for bacterial quorum sensing (Deng et al. 2011). Notably, RpfB plays a role in inhibiting the thioesterase activity of DSF synthase, facilitating the conversion of fatty acids into CoA esters. This enzyme also enhances the uptake and activation of fatty acids, thereby supporting bacterial utilization of carbon sources (Bi et al. 2014). Our findings highlight the enrichment of not only RpfB but also the associated RpfF, RpfC, and RpfG enzymes, which are involved in facilitating iron uptake (as indicated in Additional file 1: Fig. S6) (Krewulak and Vogel 2008), an essential nutrient required for bacterial growth. This suggests a potential reciprocal benefit in terms of iron availability between bacteria and plants in the rhizosphere. Intriguingly, the presence of RpfB (EC 6.2.1.3) is not confined to bacteria in the present study; it is also identified in various eukaryotic organisms, including Ascomycota, Basidiomycota, Mucoromycota, and Streptophyta (Additional file 2: Table S17). This prompts the consideration of RpfB's potential role beyond bacteria, encompassing diverse organisms within different kingdoms of life. The potential significance of RpfB in bacterial biofilm formation also exists (Additional file 1: Fig. S6). The enzyme takes part as a sensor in the processes that lead to the formation of bacterial biofilm (Additional file 1: Fig. S6), where microbes attach to surfaces, grow therein, and produce polymers that make attachment easier and matrix formation that speeds up bacterial growth and gene transcription (Donlan 2001).

Notably, quorum sensing controls the metabolism of planktonic cells that leads to the development of microbial biofilms and an increase in virulence (Preda and Săndulescu 2019). Biofilms are intricate structures composed of bacterial communities embedded within self-produced matrices. These biofilms enhance bacterial adhesion, persistence, and competition success, contributing to the colonization of surfaces and resilience in challenging environments (Flemming et al. 2016). Therefore, the enrichment of RpfB in the rhizospheric soil of *A. fruticosum* implies its potential role in regulating biofilm formation dynamics and microbial community composition within this niche.

According to the investigation conducted on the KEGG pathway labeled as 'ABC transporters' (Additional file 1: Fig. S7), a notable abundance of the gene responsible for encoding the cytoplasmic ATP-binding protein, known as RbsA (EC 3.6.3.17/7.5.2.7), was observed in the rhizospheric soil of *A. fruticosum* (Fig. 7b). This particular gene encoding the protein is present in organisms belonging to the Bacteria and Eukaryota kingdoms (Additional file 2: Table S17). This protein is a component of the tripartite RbsABC ribose transport system, which has been reported to play a role in the import of D-ribose and chemotaxis (Galloway and Furlong 1977), as well as energy coupling to the bacterial transport system (https://www.uniprot.org/uniprotkb/Q8XJX3/entry). In *Escherichia coli*, the RbsABC ribose transport complex consists of two other components: RbsC, a nucleotide binding transmembrane protein, and RbsB, a periplasmic substrate binding protein (Clifton et al. 2015). The delivered D-ribose is utilized as a precursor for nucleic acid synthesis. Generally, ATP-driven ABC transporters play a crucial role in regulating the import and export of various substances, e.g., sugars and proteins, across plasma membranes (Laub et al. 2017; Liu et al. 2019; Ye et al. 2020). Based on the number of transmembrane helices and the chemical structure of their substrates, ABC importers can be further classified into importer I and importer II (Liu et al. 2019). The ribose transporter has been reported to accommodate both of these import types (Clifton et al. 2015). The entire RbsABC complex was found to be more abundant in the rhizobiome of *A. fruticosum* in the current study compared to the bulk soil microbiome (Additional file 1: Fig. S7), with a significant enrichment of the *rbsA* gene, thus supporting the activity of this complex. It is plausible that d-ribose is present in the plant exudate to facilitate soil microbes in their nucleic acid synthesis during reproduction.

In contrast to the bulk soil, the rhizospheric soil of *A. fruticosum* exhibited a higher abundance of the gene encoding the phosphate regulon sensor histidine kinase, known as PhoR (EC 2.7.13.3) (Figs. 7b and Additional file 1: Fig. S8), within the KEGG pathway 'Two-component system'. The PhoR/PhoP two-component system, a member of the OmpR family of outer membrane proteins, controls the biosynthesis of fengycin, where PhoR serves as the sensor and PhoP as the regulator. This system operates specifically under low phosphate conditions within the medium (GUO et al. 2018). Serving as a fundamental stimulus–response coupling mechanism, the two-component regulatory system enables organisms to detect and respond to changes in various environmental conditions (Stock et al. 2000). In *Bacillus subtilis*, fengycin acts as an antifungal cyclic lipopeptide against the mold *Botrytis cinerea* (Ke et al. 2009; Vanittanakom et al. 1986). The gene encoding this enzyme is present in both bacterial and eukaryotic microbiomes of *A. fruticosum* (Additional file 2: Table S17). Hence, it is expected that this wild plant will release exudate that either lacks phosphates or contains them in minimal amounts in order to sustain the PhoR/PhoP two-component regulation system.

The KEGG pathway labeled as ‘Aminoacyl-tRNA biosynthesis’ revealed a higher abundance of the gene encoding the aspartyl-tRNA(Asn)/glutamyl-tRNA(Gln) amidotransferase subunit B, also known as GatB (EC 6.3.5.6), in the rhizospheric soil microbiome compared to the bulk soil microbiome (Figs. 7b and Additional file 1: Fig. S9). This enzyme enables microbes lacking one or both of the asparaginyl-tRNA or glutaminyl-tRNA synthetases to correctly charge Asn-tRNA (Asn) or Gln-tRNA (Gln) through the transamidation of misacylated Asp-tRNA (Asn) or Glu-tRNA (Gln) (Curnow et al. 1997). When soil microbes exhibit a deficiency in glutaminyl-tRNA synthetase, GatB experiences significant enrichment as a backup mechanism to sustain the reproductive rate, as observed in previous studies (Curnow et al. 1997; Oshikane et al. 2006). The occurrence of this reaction has been documented in *Acidithiobacillus ferrooxidans*, where the activation of phospho-Asp-tRNA(Asn) or phospho-Glu-tRNA(Gln) takes place in the presence of glutamine and ATP (Oshikane et al. 2006). In our study, we identified the gene responsible for encoding this enzyme in the archaeal, bacterial, and eukaryotic microbiomes of *A. fruticosum* (Additional file 2: Table S17). It is expected that this particular wild plant species will produce an exudate containing glutamine as a result of a mutually beneficial relationship with microbes. Additionally, ATP generation is anticipated to occur through the KEGG pathway ‘Oxidative phosphorylation,’ facilitated by the rhizobiome, as illustrated in Additional file 1: Fig. S16.

The KEGG pathway ‘Valine, leucine and isoleucine biosynthesis’ (Additional file 1: Fig. S10) reveals a higher abundance of the gene encoding acetolactate synthase I/II/III large subunit, also known as ALS (EC 2.2.1.6), in the rhizospheric soil compared to bulk soil (Fig. 7b). This dual-purpose enzyme, described by Chipman and colleagues, plays a crucial role in the initial step of valine and isoleucine synthesis (Chipman et al. 1998), involving the decarboxylation and condensation of the alpha-keto acid pyruvate (Pang et al. 2004), leading to the formation of either (S)-2-Acetolactate or (S)-2-Aceto-2-hydroxybutanoate (Additional file 1: Fig. S10). The biosynthesis of end products such as valine and isoleucine is regulated by downstream enzymes present in the two pathways, which are more enriched in the rhizospheric soil microbiome compared to the bulk soil microbiome. Previous reports suggest that the ALS enzyme is typically found in specific bacteria (Lee et al. 2015; Pang et al. 2004). However, in our study, we discovered the presence of the gene encoding this enzyme in the archaeal, bacterial, and eukaryotic microbiomes of *A. fruticosum* (Additional file 2: Table S17). Notably, the enzyme's activation relies on thiamine diphosphate and is subject to feedback inhibition, where the transcription of the encoding gene is reduced in the presence of the end-products of the two pathways, e.g., the two branched-chain amino acids (Calvo and Matthews 1994, Dailey and Cronan Jr 1986). We hypothesize that the exudate of the wild plant *A. fruticosum* might contain reasonable amount thiamine diphosphate, which is essential for the proper functioning of this enzyme.

Pyruvate dehydrogenase E1 component beta subunit, or PDHB (EC 1.2.4.1), which is highly abundant in the rhizospheric soil of *A. fruticosum*, enables pyruvate to participate in the biosynthesis of acetyl CoA via three distinct pathways: “Glycolysis/Gluconeogenesis” (Additional file 1: Fig. S12), “Citrate cycle (TCA cycle)” (Additional file 1: Fig. S13), and “Pyruvate metabolism” (Additional file 1: Fig. S14). Acetyl CoA is also produced through the action of the bidirectional acetyl-CoA C-acetyltransferase, known as ATOB (EC 2.3.1.9), in the KEGG pathway “Carbon fixation pathways in prokaryotes” (Additional file 1: Fig. S18). Numerous enzymes in these two pathways exhibit higher abundance in the rhizospheric soil of *A. fruticosum* compared to bulk soil. The ATOB enzyme also catalyzes the condensation of two acetyl-CoA molecules to form acetoacetyl-CoA (Additional file 1: Figs. S14 and S15), which represents the initial enzymatic reaction in the mevalonate (MVA) biosynthesis pathway (Dyer et al. 2009; Soto et al. 2011). Both the ‘Pyruvate metabolism’ (Additional file 1: Fig. S14) and ‘Glyoxylate and dicarboxylate metabolism’ (Additional file 1: Fig. S15) KEGG pathways highlight this direction of the chemical reaction. Moreover, the ATOB enzyme's bidirectional action is demonstrated by the KEGG pathway ‘Valine, leucine, and isoleucine degradation’ (Additional file 1: Fig. S11), where it functions as a shuttle reaction between acetoacetyl CoA and acetyl CoA. We anticipate that the two enzymes, PDHB and ATOB, will produce large amounts of the two molecules. Consequently, we predict that the rhizospheric soil microbiome of *A. fruticosum* plays a critical role in the production of acetyl CoA, which is essential for various metabolic processes such as the TCA cycle, pyruvate metabolism, and carbon fixation. The gene that codes for the enzyme ATOB in the current study was found in the archaeal, bacterial, and eukaryotic microbiomes of the wild plant *A. fruticosum*, while the enzyme PDHB was found in the bacterial and eukaryotic microbiomes (Additional file 2: Table S17).

Despite the diminished abundance of the KEGG pathway ‘Fatty acid biosynthesis’ within the rhizobiome of *A. fruticosum*, we must not overlook the significant role played by the highly prevalent enzyme, long-chain acyl-CoA synthetase (ACSL or RpfB) (EC 6.2.1.3). This enzyme facilitates communication between the aforementioned pathway (Additional file 1: Fig. S20) and the ‘Quorum sensing’ pathway (Additional file 1: Fig. S6). According to our findings, or RpfB catalyzes the conversion of acetyl CoA into long-chain acyl-CoA, a metabolite that assumes vital functions within the rhizobiome and its symbiotic plant counterpart. It serves as a reservoir of stored energy, functions as a signaling molecule in response to abiotic stress, and acts as a surface barrier or defensive compound against stressors (Zhao et al. 2021). Previous studies have also indicated its involvement in supporting ABA (abscisic acid) signaling in plants (Du et al. 2013). We posit that this metabolite may be shared between the rhizobiome and the roots of *A. fruticosum*, synergistically enhancing the plant's resilience to drought stress. Furthermore, we propose that pyruvate might be present in plant exudates to facilitate the production of acetyl CoA and acetoacetyl CoA by soil microbes, subsequently contributing to the biosynthesis of long-chain acyl-CoA as an energy source and signaling molecule in response to biotic and abiotic stresses.

Comparatively, the rhizospheric microbiome of *A. fruticosum* exhibited a greater enrichment of the KEGG pathway ‘Oxidative phosphorylation’ in contrast to the bulk soil (Additional file 1: Fig. S17). Upon comparing the rhizospheric soil microbiome of *A. fruticosum* with that of the bulk soil, we observed three highly abundant enzyme subunits (Fig. 7b). These subunits correspond to NADH-quinone oxidoreductase or complex I (EC 1.6.5.3/7.1.1.2), cytochrome c oxidase or complex IV (EC 1.9.3.1/7.1.1.9), and F-type H + -transporting ATPase or complex V (EC 3.6.3.14/7.1.2.2). Complex I, responsible for NAD + and quinol biosynthesis, facilitates the oxidation of NADH, the reduction of ubiquinone, and the transport of 4 H + /NADH across the coupling membrane (Kerscher et al. 1999). In eukaryotes, this enzyme catalyzes the initial step in various pathways of mitochondrial NADH oxidation, including ‘Oxidative phosphorylation’ (Additional file 1: Fig. S17). NAD-linked dehydrogenases from the citric acid cycle supply the enzyme, which subsequently feeds the respiratory chain. In other words, the enzyme's reaction involves the two-electron oxidation of NADH by the lipid-soluble quinone coenzyme, ubiquinone, found in the mitochondrial membrane. The complex combines the reduction of ubiquinone with the oxidation of NADH to generate a proton gradient utilized for ATP production. The genes encoding the individual proteins of complex I are present in both the cell nucleus and the mitochondrial genome (Hirst 2005). The enzyme was found in the plasma membranes of purple photosynthetic bacteria, closely related respiratory bacteria, and the mitochondria of eukaryotes. Both bacterial and eukaryotic microorganisms encode this enzyme based on our current study (Additional file 2: Table S17). Given that various substances, such as AMP and 2,4-dinitrophenol, are known to inhibit this enzyme, we postulate that the exudate of *A. fruticosum* may contain minimal to trace amounts of such substances to ensure the appropriate enrichment of this enzyme within the rhizospheric microbiome of *A. fruticosum*.

The enzyme responsible for terminating the respiratory chains of aerobic and facultative aerobic organisms is cytochrome c oxidase subunit III, or COXIII (EC 1.9.3.1/7.1.1.9), also known as complex IV, a vast transmembrane protein complex (Chan and Li 1990). Previously believed to be present in bacteria, archaea, and eukaryotic mitochondria (Castresana et al. 1994), we exclusively detected this enzyme in eukaryotic microbes in our study (Additional file 2: Table S17). COXIII possesses an intricately structured composition, comprising 13 subunits, two heme groups, and multiple metal ion cofactors (Tsukihara et al. 1996). Four cytochrome c molecules contribute one electron to complex IV, which subsequently transfers them to one oxygen molecule, four protons, and two watermolecules. In addition to binding the four protons from the inner aqueous phase, complex IV transports an additional four protons across the membrane. This elevation in the transmembrane difference of proton electrochemical potential is then harnessed by the ATP synthase to synthesize ATP (Fontanesi et al. 2006; Gladwin and Shiva 2009).

The F-type H + -transporting ATPase subunit beta, also known as complex V (EC 3.6.3.14/7.1.2.2) or ATP synthase, is a multimeric complex anchored to the mitochondrial membrane in eukaryotic cells, featuring two domains. These domains include the F0 channel domain accountable for ATP turnover in the membrane and an F1 domain extending into the lumen, which facilitates ion translocation (Alexander et al. 2021). While the enzyme also exhibits ATPase activity, it primarily relies on proton transport across the inner mitochondrial membrane to drive ATP synthesis. This enzyme orchestrates the conversion of ADP and phosphate (Pi) into ATP, utilizing the energy stored within the proton gradient across the membrane (Nelson et al. 2000). The function of this enzyme remains consistent in both prokaryotes and eukaryotes, constituting a fundamental component across all living organisms (Junge and Nelson 2015). Nevertheless, in our study, we identified the gene encoding this enzyme exclusively in bacterial and eukaryotic microorganisms within the rhizospheric soil of *A. fruticosum* (Additional file 2: Table S17).

Generally speaking, the metabolic pathway known as ‘Oxidative phosphorylation’ encompasses the enzymatic oxidation of nutrients within cells to release chemical energy, ultimately generating adenosine triphosphate (ATP). In other words, oxidative phosphorylation is a biological mechanism that utilizes oxygen reduction to synthesize adenosine triphosphate (ATP), which harbors high-energy phosphate bonds (Boyman et al. 2020). This intricate process takes place within the mitochondria of eukaryotic cells. The citric acid cycle, present in the cell, liberates the energy stored in the chemical bonds of glucose, resulting in the production of carbon dioxide and potent electron donors, namely NADH and FADH (Nath 2019). These molecules, along with oxygen, participate in oxidative phosphorylation to yield ATP, which serves as a cellular energy source (Boyman et al. 2020; Matlin 2016). Our proposition posits that the wild plant *A. fruticosum* potentially secretes glucose to fuel the oxidative phosphorylation pathway within the rhizospheric microbiome, thereby promoting the biosynthesis of ATP molecules by different complexes. This energy reservoir in the rhizobiome benefits both soil microbes and plants.

Comparatively, when analyzing the rhizospheric soil of *A. fruticosum*, we observed higher abundance of three enzyme subunits: DNA-directed RNA polymerase subunit beta’ RpB (EC 2.7.7.6), DNA polymerase III subunit delta (EC 2.7.7.7), and nucleoside-diphosphate kinase (EC 2.7.4.6), within the KEGG pathways ‘Purine metabolism’ and ‘Pyrimidine metabolism’ (Fig. 7b). These three enzymes facilitate the interaction between the aforementioned pathways. Additionally, we found a substantial presence of CMP/dCMP kinase (EC 2.7.4.25), an enzyme from the latter pathway, within the rhizobiome (Additional files 1, 2: Fig. S20 and Table S16). Notably, the two enzyme subunits DNA-directed RNA polymerase subunit beta' or RpB (EC 2.7.7.6) and DNA polymerase III subunit delta or HolA (EC 2.7.7.7) are present in members of all three kingdoms: Archaea, Bacteria, and Eukaryota. However, nucleoside-diphosphate kinase (EC 2.7.4.6) is exclusive to bacteria, while the enzyme CMP/dCMP kinase (EC 2.7.4.25) occurs in both archaea and bacteria (Additional file 2: Table S17). Previous reports suggest that an enzyme containing the RpB' subunit catalyzes the transcription of DNA into RNA using the four ribonucleoside triphosphates as substrates (Zhang et al. 1999). Conversely, the HolA subunit is responsible for DNA-template-directed extension of the 3' end of a DNA strand, albeit incapable of initiating a chain from scratch. HolA is an integral part of the core RNA polymerase complex, facilitating transcription elongation and termination but not initiation. Unlike DNA replication, transcription necessitates an additional enzyme subunit known as the sigma factor for initiation. Unfortunately, data pertaining to the abundance of the sigma factor subunit in the rhizobiome of *A. fruticosum* are lacking. Prior to DNA synthesis mediated by the HolA enzyme (EC 2.7.7.7) and RNA transcription facilitated by the RpB enzyme (EC 2.7.7.6), the enzyme nucleoside-diphosphate kinase (EC 2.7.4.6) catalyzes the transfer of terminal phosphate groups from 5′-triphosphate to 5′-diphosphate nucleotides (Additional file 1: Fig. S20). Furthermore, the enzyme CMP/dCMP kinase carries out the conversion of CMP/dCMP to CDP/dCDP and UMP/dTMP to UDP/dTDP (Bertrand et al. 2002), representing an initial step in both DNA replication and RNA transcription (Additional file 1: Fig. S20). The availability of AMP is essential for the rhizobiome to support DNA replication, which, in turn, results in diminishing AMP. Such a low level of AMP is necessary for the function of complex I within the “Oxidative phosphorylation” pathway (Additional file 1: Fig. S17).

In addition to the three enzymes involved in the oxidative phosphorylation pathway, as well as the four enzymes participating in the nucleotide metabolism pathways, our investigation revealed the presence of several other less common but highly enriched enzymes within the four aforementioned pathways in the rhizobiome. Notably, the enzyme RbsA (EC 3.6.3.17/7.5.2.7), responsible for D-ribose transport, plays a significant role in nucleotide and DNA biosynthesis. This enzyme's presence enables the rhizobiome to effectively multiply and reproduce, likely providing the plant with essential biosynthesized materials. This exemplifies the successful symbiotic relationship between plants and microbes.

The rhizosphere microbiome heavily relies on the exudates or rhizodeposits released by plant roots for its sustenance. In turn, it assists plants by facilitating nutrient uptake and recycling, as well as mitigating biotic and abiotic stresses (Devi et al. 2022; Naik et al. 2019). Low molecular weight compounds, including amino acids (e.g., glutamine), simple sugars (e.g., d-ribose and glucose), and organic acids (e.g., pyruvate), diffuse through the plant cell membrane. Conversely, high molecular weight compounds, such as proteins and polysaccharides, are transported via vesicular transport (Badri and Vivanco 2009; Badri et al. 2013). In certain circumstances, plants release amino acids into the soil as a defense strategy against pathogens, while simultaneously preventing their uptake by their own roots (Hartmann et al. 2008). Soil-dwelling bacteria also incorporate a substantial portion of these released or externally supplied amino acids into their biomass as a defense mechanism against pathogens, with the remaining portion being lost through respiration (Jones et al. 2005; Jones et al. 2009).

In conclusion, our investigation identified a variety of potentially exuded metabolites that influence the activity of the highly abundant microbes in the rhizosphere soil of A. fruticosum. We maintain that exploring the metabolic traits and characteristics of rhizosphere microbiomes associated with wild plants contributes to the discovery of novel strategies for enhancing plant growth, improving tolerance against abiotic stresses, and fortifying defense mechanisms against pathogens.

### Supplementary Information

**Additional file 1: Figure S1.** Number of genes encoding enzymes of the different functional categories and sub-categories of KEGG database across microbiomes of rhizosphere and surrounding bulk soils of A. fruticosum. Red arrows refer to subcategories investigated further.
**Figure S2.** Heatmap referring to KEGG categories in terms of gene abundance in microbiomes of rhizosphere (R) and surrounding bulk (S) soils of A. fruticosum. Red arrows refer to categories investigated further.
**Figure S3.** Heatmap referring to KEGG sub-categories in terms of gene abundance in microbiomes of rhizosphere (R) and surrounding bulk (S) soils of A. fruticosum. Red arrows refer to sub-categories investigated further.
**Figure S4.** Heatmap referring to KEGG pathways in terms of gene abundance in microbiomes of rhizosphere (R) and surrounding bulk (S) soils of A. fruticosum. Red arrows refer to pathways investigated further.
**Figure S5.** Heatmap referring to enriched enzymes of microbiomes of rhizosphere (R) and surrounding bulk (S) soils of A. fruticosum. Red arrows refer to the most enriched enzymes that were investigated further. Detailed information for the different enzymes are shown in Table S15.
**Figure S6.** KEGG pathway “Quorum sensing” of sub-category “Cellular community - prokaryotes” (category “Cellular Processes”) referring to the enriched steps at varying levels in rhizospheric microbiome of A. fruticosum. Blue arrow refers to the step with the enriched enzyme, e.g., long-chain acyl-CoA synthetase (EC 6.2.1.3), in the pathway. Colored boxes around enzyme EC or metabolite refer to the enrichment level, where red refers to the high level compared with that of the bulk soil, while blue refers to the low level compared with that of the bulk soil. See scale in the figure for intermediate enrichment levels.
**Figure S7.** KEGG pathway “ABC transporters” of sub-category “Membrane transport” (category “Environmental Information Processing”) referring to the enriched steps at varying levels in rhizospheric microbiome of A. fruticosum. Blue arrow refers to the step with the most enriched enzyme, e.g., ribose transport system ATP-binding protein (EC 3.6.3.17/7.5.2.7), in the pathway. Colored boxes around enzyme EC or metabolite refer to the enrichment level, where red refers to the high level compared with that of the bulk soil, while blue refers to the low level compared with that of the bulk soil. See scale in the figure for intermediate enrichment levels.
**Figure S8.** KEGG pathway “Two-component system” of sub-category “Signal transduction” (category “Environmental Information Processing”) referring to the enriched steps at varying levels in rhizospheric microbiome of A. fruticosum. Blue arrow refers to the step with the most enriched enzyme, e.g., phosphate regulon sensor histidine kinase PhoR (EC 2.7.13.3), in the pathway. Colored boxes around enzyme EC or metabolite refer to the enrichment level, where red refers to the high level compared with that of the bulk soil, while blue refers to the low level compared with that of the bulk soil. See scale in the figure for intermediate enrichment levels.
**Figure S9.** KEGG pathway “Aminoacyl-tRNA biosynthesis” of sub-category “Translation” (category “Genetic Information Processing”) referring to ECs of the enriched enzymes at varying levels in rhizospheric microbiome of A. fruticosum. Blue arrow refers to the step with the most enriched enzyme, e.g., aspartyl-tRNA(Asn)/glutamyltRNA( Gln) amidotransferase subunit B (EC 6.3.5.6/6.3.5.7), in the pathway. Colored boxes around enzyme EC or metabolite refer to the enrichment level, where red refers to the high level compared with that of the bulk soil, while blue refers to the low level compared with that of the bulk soil. See scale in the figure for intermediate enrichment levels.
**Figure S10.** KEGG pathway “Valine, leucine and isoleucine biosynthesis” of sub-category “Amino acids metabolism” (category “Metabolism”) referring to ECs of the enriched enzymes at varying levels in rhizospheric microbiome of A. fruticosum. Blue arrow refers to the step with the most enriched enzyme, e.g., acetolactate synthase I/II/III large subunit (EC 2.2.1.6), in the pathway. Colored boxes around enzyme EC or metabolite refer to the enrichment level, where red refers to the high level compared with that of the bulk soil, while blue refers to the low level compared with that of the bulk soil. See scale in the figure for intermediate enrichment levels.
**Figure S11.** KEGG pathway “Valine, leucine and isoleucine degradation” of sub-category “Amino acids metabolism” (category “Metabolism”) referring to ECs of the enriched enzymes at varying levels in rhizospheric microbiome of A. fruticosum. Blue arrow refers to the step with the most enriched enzyme, e.g., acetyl-CoA C-acetyltransferase (EC 2.3.1.9), in the pathway. Colored boxes around enzyme EC or metabolite refer to the enrichment level, where red refers to the high level compared with that of the bulk soil, while blue refers to the low level compared with that of the bulk soil. See scale in the figure for intermediate enrichment levels.
**Figure S12.** KEGG pathway “Glycolysis / Gluconeogenesis” of sub-category “Carbohydrate metabolism” (category “Metabolism”) referring to ECs of the enriched enzymes at varying levels in rhizospheric microbiome of A. fruticosum. Blue arrow refers to the step with the most enriched enzyme, e.g., pyruvate dehydrogenase E1 component beta subunit (EC 1.2.4.1), in the pathway. Colored boxes around enzyme EC or metabolite refer to the enrichment level, where red refers to the high level compared with that of the bulk soil, while blue refers to the low level compared with that of the bulk soil. See scale in the figure for intermediate enrichment levels.
**Figure S13.** KEGG pathway “Citrate cycle (TCA cycle)” of sub-category “Carbohydrate metabolism” (category “Metabolism”) referring to ECs of the enriched enzymes at varying levels in rhizospheric microbiome of A. fruticosum. Blue arrow refers to the step with the most enriched enzyme, e.g., pyruvate dehydrogenase E1 component beta subunit (EC 1.2.4.1), in the pathway. Colored boxes around enzyme EC or metabolite refer to the enrichment level, where red refers to the high level compared with that of the bulk soil, while blue refers to the low level compared with that of the bulk soil. See scale in the figure for intermediate enrichment levels.
**Figure S14.** KEGG pathway “Pyruvate metabolism” of sub-category “Carbohydrate metabolism” (category “Metabolism”) referring to ECs of the enriched enzymes at varying levels in rhizospheric microbiome of A. fruticosum. Blue arrows refer to the steps with the most enriched enzymes, e.g., acetyl-CoA C-acetyltransferase (EC 2.3.1.9) and pyruvate dehydrogenase E1 component beta subunit (EC 1.2.4.1), in the pathway. Colored boxes around enzyme EC or metabolite refer to the enrichment level, where red refers to the high level compared with that of the bulk soil, while blue refers to the low level compared with that of the bulk soil. See scale in the figure for intermediate enrichment levels.
**Figure S15.** KEGG pathway “Glyoxylate and dicarboxylate metabolism” of sub-category “Carbohydrate metabolism” (category “Metabolism”) referring to ECs of the enriched enzymes at varying levels in rhizospheric microbiome of A. fruticosum. Blue arrow refers to the step with the most enriched enzyme, e.g., acetyl-CoA Cacetyltransferase (EC 2.3.1.9), in the pathway. Colored boxes around enzyme EC or metabolite refer to the enrichment level, where red refers to the high level compared with that of the bulk soil, while blue refers to the low level compared with that of the bulk soil. See scale in the figure for intermediate enrichment levels.
**Figure S16.** KEGG pathway “Oxidative phosphorylation” of sub-category “Energy metabolism” (category “Metabolism”) referring to ECs of the enriched enzymes at varying levels in rhizospheric microbiome of A. fruticosum. Blue arrows refer to the steps with the most enriched enzymes, e.g., NADH-quinone oxidoreductase subunit J (EC 1.6.5.3/7.1.1.2), cytochrome c oxidase subunit III (EC 1.9.3.1/7.1.1.9) and F-type H+-transporting ATPase subunit beta (EC 3.6.3.14/7.1.2.2), in the pathway. Colored boxes around enzyme EC or metabolite refer to the enrichment level, where red refers to the high level compared with that of the bulk soil, while blue refers to the low level compared with that of the bulk soil. See scale in the figure for intermediate enrichment levels.
**Figure S17.** KEGG pathway “Carbon fixation pathways in prokaryotes” of sub-category “Energy metabolism” (category “Metabolism”) referring to ECs of the enriched enzymes at varying levels in rhizospheric microbiome of A. fruticosum. Blue arrow refers to the step with the most enriched enzyme, e.g., acetyl-CoA C-acetyltransferase (EC 2.3.1.9), in the pathway. Colored boxes around enzyme EC or metabolite refer to the enrichment level, where red refers to the high level compared with that of the bulk soil, while blue refers to the low level compared with that of the bulk soil. See scale in the figure for intermediate enrichment levels.
**Figure S18.** KEGG pathway “Purine metabolism” of sub-category “Nucleotide metabolism” (category “Metabolism”) referring to ECs of the enriched enzymes at varying levels in rhizospheric microbiome of A. fruticosum. Blue arrows refer to the steps with the most enriched enzymes, e.g., DNA-directed RNA polymerase subunit beta' (EC 2.7.7.6), DNA polymerase III subunit delta (EC 2.7.7.7) and nucleoside-diphosphate kinase (EC 2.7.4.6), in the pathway. Colored boxes around enzyme EC or metabolite refer to the enrichment level, where red refers to the highest and blue refers to the lowest. See scale in the figure for intermediate enrichment levels.
**Figure S19.** KEGG pathway “Pyrimidine metabolism” of sub-category “Nucleotide metabolism” (category “Metabolism”) referring to ECs of the enriched enzymes at varying levels in rhizospheric microbiome of A. fruticosum. Blue arrows refer to the steps with the most enriched enzymes, e.g., DNA-directed RNA polymerase subunit beta' (EC 2.7.7.6), DNA polymerase III subunit delta (EC 2.7.7.7), nucleoside-diphosphate kinase (EC 2.7.4.6), carbamoyl-phosphate synthase large subunit (EC 6.3.5.5) and CMP/dCMP kinase (EC 2.7.4.25), in the pathway. Colored boxes around enzyme EC or metabolite refer to the enrichment level, where red refers to the high level compared with that of the bulk soil, while blue refers to the low level compared with that of the bulk soil. See scale in the figure for intermediate enrichment levels.
**Figure S20.** The less enriched KEGG pathway “Fatty acid biosynthesis” of sub-category “Lipid metabolism” (category “Metabolism”) that refers to the highly enriched enzyme, e.g., long-chain acyl-CoA synthetase (EC 6.2.1.3). Colored boxes around enzyme EC or metabolite refer to the enrichment level, where red refers to the high level compared with that of the bulk soil, while blue refers to the low level compared with that of the bulk soil.

**Additional file 2: Table S1.** Sequence alignment results of gene queries of microbiomes of rhizosphere (R) and surrounding bulk (S) soils of A. fruticosum. Query ID refers to genes in individual or mixed less abundant microbiome samples, while subject IDs refers to the sequences in the National Center for Biotechnology Information (NCBI) that showed considerable sequence homology with one or more gene queries.
**Table S2.** Number of genes encoding enzymes of different functional categories of Kyoto Encyclopedia of Genes and Genomes (KEGG) level 1 across microbiomes of rhizosphere (R) and surrounding bulk (S) soils of A. fruticosum. Gray color boxes refer to less frequent (˂ 50,000) KEGG categories that were not analyzed further.
**Table S3.** Abundance of assembled genes encoding enzymes of different functional categories of Kyoto Encyclopedia of Genes and Genomes (KEGG) level 1 in microbiomes of rhizosphere (R) and surrounding bulk (S) soils of A. fruticosum. Gray color boxes refer to less frequent (˂ 50,000) KEGG categories that were not analyzed further.
**Table S4.** Number of genes encoding enzymes of highly enriched functional sub-categories of KEGG level 2 across microbiomes of rhizosphere (R) and surrounding bulk (S) soils of A. fruticosum. Text in gray refers to less frequent (˂ 10,000) KEGG sub-categories that were not analyzed further.
**Table S5.** Highly enriched functional categories and their sub-categories of Kyoto Encyclopedia of Genes and Genomes (KEGG) functional orthologs (KO) database in microbiomes of A. fruticosum. Boxes with gray color either refer to less KEGG sub-categories with less enriched enzymes and were not analyzed further.
**Table S6.** Abundance of assembled genes encoding enzymes of highly enriched functional sub-categories of KEGG level 2 in microbiomes of rhizosphere (R) and surrounding bulk (S) soils of A. fruticosum.
**Table S7.** Abundance of genes encoding enzymes of highly enriched functional sub-categories of KEGG level 2 in microbiomes of rhizosphere (R) and surrounding bulk (S) soils of A. fruticosum.
**Table S8.** Number of genes encoding enzymes of highly enriched functional KEGG pathways (level 3) across microbiomes of rhizosphere (R) and surrounding bulk (S) soils of A. fruticosum. Text in gray refers to less frequent (< 14,000) KEGG sub-categories that were not analyzed further.
**Table S9.** Highly enriched KEGG pathways in microbiomes of A. fruticosum. Gray boxes refer to pathways with less enriched enzymes that were nor analyzed further.
**Table S10.** Abundance of genes encoding enzymes of highly enriched functional KEGG pathways in microbiomes of rhizosphere (R) and surrounding bulk (S) soils of A. fruticosum. Text in gray refers to less frequent (< 14,000) KEGG sub-categories that were not analyzed further.
**Table S11.** Abundance of genes encoding enzymes of highly enriched KEGG pathways in microbiomes of rhizosphere (R) and surrounding bulk (S) soils of A. fruticosum. Blue box = Cellular Processes category, red box = Environmental Information Processing category, yellow box = Genetic Information Processing category, green box = Metabolism category, gray box = pathways with less enriched enzymes that were not analyzed further.
**Table S12.** Number of assembled genes encoding enzymes of Kyoto Encyclopedia of Genes and Genomes (KEGG) functional orthologs (KO) database across microbiomes of rhizosphere (R) and surrounding bulk (S) soils of A. fruticosum. Text in gray refers to less enriched (< 1,400) enzymes that were not analyzed further. Full description of enzymes along with their KEGG pathways are shown in Table S15.
**Table S13.** Abundance of assembled genes encoding enzymes of Kyoto Encyclopedia of Genes and Genomes (KEGG) functional orthologs (KO) database in microbiomes of rhizosphere (R) and surrounding bulk (S) soils of A. fruticosum. Full description of enzymes along with their KEGG pathways are shown in Table S15.
**Table S14.** Abundance of assembled genes encoding the most enriched enzymes of Kyoto Encyclopedia of Genes and Genomes (KEGG) functional orthologs (KO) database in microbiomes of rhizosphere (R) and surrounding bulk (S) soils of A. fruticosum. Full description of enzymes along with their KEGG pathways are shown in Table S15.
**Table S15.** Annotation results of gene queries of microbiomes of rhizosphere and surrounding bulk soils of A. fruticosum in terms of Kyoto Encyclopedia of Genes and Genomes (KEGG) functional orthologs (KO) ID, definition along with enzyme commission (ECs) and their KEGG pathways.
**Table S16.** Highly enriched KEGG levels with the most highly enriched enzymes in microbiomes of A. fruticosum. Gray boxes refer to less frequent KEGG pathways that were not analyzed further.
**Table S17.** Taxonomic prediction of microbes encoding enzymes in microbiomes of rhizosphere and surrounding bulk soils of A. fruticosum along with their KEGG functional pathways and ortholog (KO) IDs.

## Data Availability

The Supplementary Material for this article can be found online at:

## References

[CR1] Al-Eisawi DM, Al-Ruzayza S (2015). The flora of holy Mecca district, Saudi Arabia. Intl J Biodivers Conserv.

[CR2] Alexander SP, Kelly E, Mathie A, Peters JA, Veale EL, Armstrong JF, Faccenda E, Harding SD, Pawson AJ, Southan C, Davies JA, Amarosi L, Anderson CMH, Beart PM, Broer S, Dawson PA, Hagenbuch B, Hammond JR, Inui KI, Kanai Y (2021). The concise guide to pharmacology 2021/22:Transporters. Br J Pharmacol.

[CR3] Alzahrani DA (2021). Complete chloroplast genome of *Abutilon fruticosum*: genome structure, comparative and phylogenetic analysis. Plants.

[CR4] Badri D, Vivanco J (2009). Regulation and function of root exudates. Plant Cell Environ.

[CR5] Badri DV, Chaparro JM, Zhang R, Shen Q, Vivanco JM (2013). Application of natural blends of phytochemicals derived from the root exudates of *Arabidopsis* to the soil reveal that phenolic-related compounds predominantly modulate the soil microbiome. J Biol Chem.

[CR6] Berendsen RL, Pieterse CM, Bakker PA (2012). The rhizosphere microbiome and plant health. Trends Plant Sci.

[CR7] Bertrand T, Briozzo P, Assairi L, Ofiteru A, Bucurenci N, Munier-Lehmann H, Golinelli-Pimpaneau B, Bârzu O, Gilles A-M (2002). Sugar specificity of bacterial CMP kinases as revealed by crystal structures and mutagenesis of *Escherichia coli* enzyme. J Mol Biol.

[CR8] Bi H, Yu Y, Dong H, Wang H, Cronan JE (2014). *Xanthomonas campestris* RpfB is a fatty Acyl-CoA ligase required to counteract the thioesterase activity of the RpfF diffusible signal factor (DSF) synthase. Mol Microbiol.

[CR9] Boyman L, Karbowski M, Lederer WJ (2020). Regulation of mitochondrial ATP production:Ca(2+) signaling and quality control. Trends Mol Med.

[CR10] Bulgarelli D, Garrido-Oter R, Munch PC, Weiman A, Droge J, Pan Y, McHardy AC, Schulze-Lefert P (2015). Structure and function of the bacterial root microbiota in wild and domesticated barley. Cell Host Microbe.

[CR11] Calvo JM, Matthews RG (1994). The leucine-responsive regulatory protein, a global regulator of metabolism in *Escherichia coli*. Microbiol Rev.

[CR12] Carlton JM, Angiuoli SV, Suh BB, Kooij TW, Pertea M, Silva JC, Ermolaeva MD, Allen JE, Selengut JD, Koo HL, Peterson JD, Pop M, Kosack DS, Shumway MF, Bidwell SL, Shallom SJ, van Aken SE, Riedmuller SB, Feldblyum TV, Cho JK, Quackenbush J, Sedegah M, Shoaibi A, Cummings LM, Florens L, Yates JR, Raine JD, Sinden RE, Harris MA, Cunningham DA, Preiser PR, Bergman LW, Vaidya AB, Van Lin LH, Janse CJ, Waters AP, Smith MO, White OR, Salzberg SL, Venter JC, Fraser CM, Hoffman SL, Gardner MJ, Carucci DJ (2002). Genome sequence and comparative analysis of the model rodent malaria parasite *Plasmodium yoelii yoelii*. Nature.

[CR13] Castresana J, Lübben M, Saraste M, Higgins D (1994). Evolution of cytochrome oxidase, an enzyme older than atmospheric oxygen. EMBO J.

[CR99] Chan SI, Li PM (1990) Cytochrome c oxidase: Understanding nature’s design of a proton pump. Biochemistry. 10.1021/bi00453a001 10.1021/bi00453a001 2157476

[CR14] Chen C, Zhou Y, Fu H, Xiong X, Fang S, Jiang H, Wu J, Yang H, Gao J, Huang L (2021). Expanded catalog of microbial genes and metagenome-assembled genomes from the pig gut microbiome. Nat Commun.

[CR15] Chipman D, Barak Z, Schloss JV (1998). Biosynthesis of 2-aceto-2-hydroxy acids:acetolactate synthases and acetohydroxyacid synthases. Biochim Biophys Acta.

[CR16] Claesson MJ, Wang Q, O'Sullivan O, Greene-Diniz R, Cole JR, Ross RP, O'Toole PW (2010). Comparison of two next-generation sequencing technologies for resolving highly complex microbiota composition using tandem variable 16S rRNA gene regions. Nucleic Acids Res.

[CR17] Clifton MC, Simon MJ, Erramilli SK, Zhang H, Zaitseva J, Hermodson MA, Stauffacher CV (2015). *In vitro* reassembly of the ribose ATP-binding cassette transporter reveals a distinct set of transport complexes. J Biol Chem.

[CR18] Curnow AW, Hong K, Yuan R, Kim S, Martins O, Winkler W, Henkin TM, Soll D (1997). Glu-tRNAGln amidotransferase:a novel heterotrimeric enzyme required for correct decoding of glutamine codons during translation. Proc Natl Acad Sci USA.

[CR19] Dailey F, Cronan J (1986). Acetohydroxy acid synthase I, a required enzyme for isoleucine and valine biosynthesis in *Escherichia coli* K-12 during growth on acetate as the sole carbon source. J Bacteriol.

[CR20] Deng Y, Wu J, Tao F, Zhang LH (2011). Listening to a new language:DSF-based quorum sensing in Gram-negative bacteria. Chem Rev.

[CR21] DeSantis TZ, Hugenholtz P, Larsen N, Rojas M, Brodie EL, Keller K, Huber T, Dalevi D, Hu P, Andersen GL (2006). Greengenes, a chimera-checked 16S rRNA gene database and workbench compatible with ARB. Appl Environ Microbiol.

[CR22] Devi R, Kaur T, Kour D, Yadav A, Yadav AN, Suman A, Ahluwalia AS, Saxena AK (2022). Minerals solubilizing and mobilizing microbiomes: a sustainable approaches for managing minerals deficiency in agricultural soil. J Appl Microbiol.

[CR23] Dilthey AT, Jain C, Koren S, Phillippy AM (2019). Strain-level metagenomic assignment and compositional estimation for long reads with MetaMaps. Nat Commun.

[CR24] Donlan RM (2001). Biofilm formation:a clinically relevant microbiological process. Clin Infect Dis.

[CR25] Doyle JJ, Doyle JL (1987). A rapid DNA isolation procedure for small quantities of fresh leaf tissue. Phytochem Bull.

[CR26] Du ZY, Chen MX, Chen QF, Xiao S, Chye ML (2013). Overexpression of *Arabidopsis* acyl-CoA-binding protein ACBP2 enhances drought tolerance. Plant Cell Environ.

[CR27] Dyer JH, Maina A, Gomez ID, Cadet M, Oeljeklaus S, Schiedel AC (2009). Cloning, expression and purification of an acetoacetyl CoA thiolase from sunflower cotyledon. Int J Biol Sci.

[CR28] Flemming HC, Wingender J, Szewzyk U, Steinberg P, Rice SA, Kjelleberg S (2016). Biofilms: an emergent form of bacterial life. Nat Rev Microbiol.

[CR29] Fontanesi F, Soto IC, Horn D, Barrientos A (2006). Assembly of mitochondrial cytochrome c-oxidase, a complicated and highly regulated cellular process. Am J Physiol Cell Physiol.

[CR30] Fu L, Niu B, Zhu Z, Wu S, Li W (2012). CD-HIT: accelerated for clustering the next-generation sequencing data. Bioinform.

[CR31] Galloway DR, Furlong CE (1977). The role of ribose-binding protein in transport and chemotaxis in *Escherichia coli* K12. Arch Biochem Biophys.

[CR32] Gladwin MT, Shiva S (2009). The ligand binding battle at cytochrome c oxidase:how NO regulates oxygen gradients in tissue. Circ Res.

[CR33] Gouda HM, Morsy AA, Youssef AK, Tolba IAE-M, Hassan GOO (2022). Phytochemical profile and antimicrobial assessment of *Abutilon fruticosum* Guill. & Perr. growing in Gebel Elba, Egypt. Egypt J Chem.

[CR34] Guo Q-G, Dong L-H, Wang P-P, Li S-Z, Zhao W-S, Lu X-Y, Zhang X-Y, Ping M (2018). The PhoR/PhoP two-component system regulates fengycin production in *Bacillus subtilis* NCD-2 under low-phosphate conditions. J Integr Agric.

[CR35] Hartman K, van der Heijden MG, Roussely-Provent V, Walser JC, Schlaeppi K (2017). Deciphering composition and function of the root microbiome of a legume plant. Microbiome.

[CR36] Hartmann A, Rothballer M, Schmid M (2008). Lorenz Hiltner, a pioneer in rhizosphere microbial ecology and soil bacteriology research. Plant Soil.

[CR37] Hirst J (2005). Energy transduction by respiratory complex I—an evaluation of current knowledge. Biochem Soc Trans.

[CR38] Holden N (2019). You are what you can find to eat:bacterial metabolism in the rhizosphere. Curr Issues Mol Biol.

[CR39] Huang X-F, Chaparro JM, Reardon KF, Zhang R, Shen Q, Vivanco JM (2014). Rhizosphere interactions:root exudates, microbes, and microbial communities. Botany.

[CR40] Hurt RA, Qiu X, Wu L, Roh Y, Palumbo AV, Tiedje JM, Zhou J (2001). Simultaneous recovery of RNA and DNA from soils and sediments. Appl Environ Microbiol.

[CR41] Huson DH, Mitra S, Ruscheweyh H-J, Weber N, Schuster SC (2011). Integrative analysis of environmental sequences using MEGAN4. Genome Res.

[CR42] Huson DH, Beier S, Flade I, Górska A, El-Hadidi M, Mitra S, Ruscheweyh H-J, Tappu R (2016). MEGAN community edition-interactive exploration and analysis of large-scale microbiome sequencing data. PLoS Comput Biol.

[CR43] Jones D, Kemmitt S, Wright D, Cuttle S, Bol R, Edwards A (2005). Rapid intrinsic rates of amino acid biodegradation in soils are unaffected by agricultural management strategy. Soil Biol Biochem.

[CR44] Jones DL, Nguyen C, Finlay RD (2009). Carbon flow in the rhizosphere:carbon trading at the soil–root interface. Plant Soil.

[CR45] Junge W, Nelson N (2015). ATP synthase. Annu Rev Biochem.

[CR46] Kanehisa M, Goto S, Hattori M, Aoki-Kinoshita KF, Itoh M, Kawashima S, Katayama T, Araki M, Hirakawa M (2006). From genomics to chemical genomics:new developments in KEGG. Nucleic Acids Res.

[CR47] Kanehisa M, Goto S, Sato Y, Kawashima M, Furumichi M, Tanabe M (2014). Data, information, knowledge and principle:back to metabolism in KEGG. Nucleic Acids Res.

[CR48] Kanehisa M, Sato Y, Kawashima M, Furumichi M, Tanabe M (2016). KEGG as a reference resource for gene and protein annotation. Nucleic Acids Res.

[CR49] Kanehisa M, Sato Y, Morishima K (2016). BlastKOALA and GhostKOALA: KEGG tools for functional characterization of genome and metagenome sequences. J Mol Biol.

[CR50] Karlsson FH, Fåk F, Nookaew I, Tremaroli V, Fagerberg B, Petranovic D, Bäckhed F, Nielsen J (2012). Symptomatic atherosclerosis is associated with an altered gut metagenome. Nat Commun.

[CR51] Karlsson FH, Tremaroli V, Nookaew I, Bergstrom G, Behre CJ, Fagerberg B, Nielsen J, Backhed F (2013). Gut metagenome in European women with normal, impaired and diabetic glucose control. Nature.

[CR52] Ke WJ, Chang BY, Lin TP, Liu ST (2009). Activation of the promoter of the fengycin synthetase operon by the UP element. J Bacteriol.

[CR53] Kennedy NA, Walker AW, Berry SH, Duncan SH, Farquarson FM, Louis P, Thomson JM, Satsangi J, Flint HJ (2014). The impact of different DNA extraction kits and laboratories upon the assessment of human gut microbiota composition by 16S rRNA gene sequencing. PLoS ONE.

[CR54] Kerscher SJ, Okun JG, Brandt U (1999). A single external enzyme confers alternative NADH:ubiquinone oxidoreductase activity in Yarrowia lipolytica. J Cell Sci.

[CR55] Kolton M, Green SJ, Harel YM, Sela N, Elad Y, Cytryn E (2012). Draft genome sequence of *Flavobacterium* sp. strain F52, isolated from the rhizosphere of bell pepper (*Capsicum annuum* L. cv. *Maccabi*). J Bacteriol.

[CR56] Krewulak KD, Vogel HJ (2008). Structural biology of bacterial iron uptake. Biochim Biophys Acta.

[CR57] Laub KR, Marek M, Stanchev LD, Herrera SA, Kanashova T, Bourmaud A, Dittmar G, Gunther Pomorski T (2017). Purification and characterisation of the yeast plasma membrane ATP binding cassette transporter Pdr11p. PLoS ONE.

[CR58] Lee S-C, Jung I-P, Baig IA, Chien PN, La I-J, Yoon M-Y (2015). Mutational analysis of critical residues of FAD-independent catabolic acetolactate synthase from *Enterococcus faecalis* V583. Intl J Biol Macromol.

[CR59] Li W, Godzik A (2006). Cd-hit:a fast program for clustering and comparing large sets of protein or nucleotide sequences. Bioinform.

[CR60] Li J, Jia H, Cai X, Zhong H, Feng Q, Sunagawa S, Arumugam M, Kultima JR, Prifti E, Nielsen T, Juncker AS, Manichanh C, Chen B, Zhang W, Levenez F, Wang J, Xu X, Xiao L, Liang S, Zhang D, Zhang Z, Chen W, Zhao H, Al-Aama JY, Edris S, Yang J, Wang J, Hansen T, Nielsen HB, Brunak S, Kristiansen K, Guarner F, Pedersen O, Doré J, Ehrlich SD, Bork P, Wang J (2014). An integrated catalog of reference genes in the human gut microbiome. Nat Biotechnol.

[CR61] Liu H, Cheng M, Zhao S, Lin C, Song J, Yang Q (2019). ATP-binding cassette transporter regulates N, N′-diacetylchitobiose transportation and chitinase production in* Trichoderma asperellu*m T4. Int J Mol Sci.

[CR62] Matlin KS (2016). The heuristic of form: mitochondrial morphology and the explanation of oxidative phosphorylation. J Hist Biol.

[CR63] Mende DR, Waller AS, Sunagawa S, Järvelin AI, Chan MM, Arumugam M, Raes J, Bork P (2012). Assessment of metagenomic assembly using simulated next generation sequencing data. PLoS ONE.

[CR64] Naik K, Mishra S, Srichandan H, Singh PK, Sarangi PK (2019). Plant growth promoting microbes: potential link to sustainable agriculture and environment. Biocatal Agric Biotechnol.

[CR65] Nath S (2019). Integration of demand and supply sides in the ATP energy economics of cells. Biophys Chem.

[CR66] Nelson N, Perzov N, Cohen A, Hagai K, Padler V, Nelson H (2000). The cellular biology of proton-motive force generation by V-ATPases. J Exp Biol.

[CR67] Nielsen HB, Almeida M, Juncker AS, Rasmussen S, Li J, Sunagawa S, Plichta DR, Gautier L, Pedersen AG, Le Chatelier E (2014). Identification and assembly of genomes and genetic elements in complex metagenomic samples without using reference genomes. Nat Biotechnol.

[CR68] Oh J, Byrd AL, Deming C, Conlan S, Program NCS, Kong HH, Segre JA (2014). Biogeography and individuality shape function in the human skin metagenome. Nature.

[CR69] Oshikane H, Sheppard K, Fukai S, Nakamura Y, Ishitani R, Numata T, Sherrer RL, Feng L, Schmitt E, Panvert M, Blanquet S, Mechulam Y, Soll D, Nureki O (2006). Structural basis of RNA-dependent recruitment of glutamine to the genetic code. Science.

[CR70] Pang SS, Duggleby RG, Schowen RL, Guddat LW (2004). The crystal structures of *Klebsiella pneumoniae* acetolactate synthase with enzyme-bound cofactor and with an unusual intermediate. J Biol Chem.

[CR71] Patel MK, Rajput AP (2013). Therapeutic significance of *Abutilon indicum*: an overview. Am J Pharm Tech Res.

[CR72] Pérez-Jaramillo JE, Carrión VJ, Bosse M, Ferrão LF, De Hollander M, Garcia AA, Ramírez CA, Mendes R, Raaijmakers JM (2017). Linking rhizosphere microbiome composition of wild and domesticated *Phaseolus vulgaris* to genotypic and root phenotypic traits. ISME J.

[CR73] Pett-Ridge J, Firestone MK (2017). Using stable isotopes to explore root-microbe-mineral interactions in soil. Rhizosphere.

[CR74] Pett-Ridge J, Shi S, Estera-Molina K, Nuccio E, Yuan M, Rijkers R, Swenson T, Zhalnina K, Northen T, Zhou J, Gupta VVSR, Sharma AK (2021). Rhizosphere carbon turnover from cradle to grave: the role of microbe–plant interactions. Rhizosphere biology: interactions between microbes and plants.

[CR75] Preda VG, Săndulescu O (2019). Communication is the key: biofilms, quorum sensing, formation and prevention. Discoveries.

[CR76] Quince C, Walker AW, Simpson JT, Loman NJ, Segata N (2017). Shotgun metagenomics, from sampling to analysis. Nat Biotechnol.

[CR77] Raes J, Foerstner KU, Bork P (2007). Get the most out of your metagenome:computational analysis of environmental sequence data. Curr Opin Microbiol.

[CR78] Rudrappa T, Bais HP (2008). Curcumin, a known phenolic from *Curcuma longa*, attenuates the virulence of *Pseudomonas aeruginosa* PAO1 in whole plant and animal pathogenicity models. J Agric Food Chem.

[CR79] Rutherford ST, Bassler BL (2012). Bacterial quorum sensing:its role in virulence and possibilities for its control. Cold Spring Harb Perspect Med.

[CR80] Saleem M, Law AD, Sahib MR, Pervaiz ZH, Zhang Q (2018). Impact of root system architecture on rhizosphere and root microbiome. Rhizosphere.

[CR81] Schlaeppi K, Dombrowski N, Oter RG, Loren V, van Themaat E, Schulze-Lefert P (2014). Quantitative divergence of the bacterial root microbiota in *Arabidopsis thaliana* relatives. Proc Natl Acad Sci USA.

[CR82] Scholz M, Ward DV, Pasolli E, Tolio T, Zolfo M, Asnicar F, Truong DT, Tett A, Morrow AL, Segata N (2016). Strain-level microbial epidemiology and population genomics from shotgun metagenomics. Nat Methods.

[CR83] Segata N, Izard J, Waldron L, Gevers D, Miropolsky L, Garrett WS, Huttenhower C (2011). Metagenomic biomarker discovery and explanation. Genome Biol.

[CR84] Soto G, Stritzler M, Lisi C, Alleva K, Pagano ME, Ardila F, Mozzicafreddo M, Cuccioloni M, Angeletti M, Ayub ND (2011). Acetoacetyl-CoA thiolase regulates the mevalonate pathway during abiotic stress adaptation. J Exp Bot.

[CR85] Stewart RD, Auffret MD, Warr A, Wiser AH, Press MO, Langford KW, Liachko I, Snelling TJ, Dewhurst RJ, Walker AW, Roehe R, Watson M (2018). Assembly of 913 microbial genomes from metagenomic sequencing of the cow rumen. Nat Commun.

[CR86] Stock AM, Robinson VL, Goudreau PN (2000). Two-component signal transduction. Annu Rev Biochem.

[CR87] Sugiyama A (2019). The soybean rhizosphere: metabolites, microbes, and beyond—a review. J Adv Res.

[CR88] Sugiyama A, Ueda Y, Zushi T, Takase H, Yazaki K (2014). Changes in the bacterial community of soybean rhizospheres during growth in the field. PLoS ONE.

[CR89] Suryawanshi VS, Umate SR (2020). A Review on phytochemical constituents of *Abutilon indicum* (Link) sweet-an important medicinal plant in Ayurveda. Pla Sci.

[CR90] Tringe SG, von Mering C, Kobayashi A, Salamov AA, Chen K, Chang HW, Podar M, Short JM, Mathur EJ, Detter JC, Bork P, Hugenholtz P, Rubin EM (2005). Comparative metagenomics of microbial communities. Science.

[CR91] Tsukihara T, Aoyama H, Yamashita E, Tomizaki T, Yamaguchi H, Shinzawa-Itoh K, Nakashima R, Yaono R, Yoshikawa S (1996). The whole structure of the 13-subunit oxidized cytochrome c oxidase at 2.8 Å. Science.

[CR92] Vanittanakom N, Loeffler W, Koch U, Jung G (1986). Fengycin–a novel antifungal lipopeptide antibiotic produced by *Bacillus subtilis* F-29-3. J Antibiot.

[CR93] Wilkins LG, Ettinger CL, Jospin G, Eisen JA (2019). Metagenome-assembled genomes provide new insight into the microbial diversity of two thermal pools in Kamchatka, Russia. Sci Rep.

[CR94] Ye Z, Lu Y, Wu T (2020). The impact of ATP-binding cassette transporters on metabolic diseases. Nutr Metab.

[CR95] Yin C, Hulbert SH, Schroeder KL, Mavrodi O, Mavrodi D, Dhingra A, Schillinger WF, Paulitz TC (2013). Role of bacterial communities in the natural suppression of *Rhizoctonia solani* bare patch disease of wheat (*Triticum aestivum* L.). Appl Environ Microbiol.

[CR96] Zachow C, Müller H, Tilcher R, Berg G (2014). Differences between the rhizosphere microbiome of *Beta vulgaris* ssp. maritima—ancestor of all beet crops—and modern sugar beets. Front Microbiol.

[CR97] Zhang G, Campbell EA, Minakhin L, Richter C, Severinov K, Darst SA (1999). Crystal structure of *Thermus aquaticus* core RNA polymerase at 3.3 Å resolution. Cell.

[CR98] Zhao Y, Fu W, Hu C, Chen G, Xiao Z, Chen Y, Wang Z, Cheng H (2021). Variation of rhizosphere microbial community in continuous mono-maize seed production. Sci Rep.

