# Supplementary material for: Exploration of genes encoding KEGG pathway enzymes in rhizospheric microbiome of the wild plant Abutilon fruticosum
Source: AMB Express. 2024 Feb 21;14:27. doi: 10.1186/s13568-024-01678-4 (PMC10881953; doi:10.1186/s13568-024-01678-4)
Supplement: Supplementary file 1 — Additional file 1: Figure S1. Number of genes encoding enzymes of the different functional categories and sub-categories of KEGG database across microbiomes of rhizosphere and surrounding bulk soils of A. fruticosum. Red arrows refer to subcategories investigated further. Figure S2. Heatmap referring to KEGG categories in terms of gene abundance in microbiomes of rhizosphere (R) and surrounding bulk (S) soils of A. fruticosum. Red arrows refer to categories investigated further. Figure S3. Heatmap referring to KEGG sub-categories in terms of gene abundance in microbiomes of rhizosphere (R) and surrounding bulk (S) soils of A. fruticosum. Red arrows refer to sub-categories investigated further. Figure S4. Heatmap referring to KEGG pathways in terms of gene abundance in microbiomes of rhizosphere (R) and surrounding bulk (S) soils of A. fruticosum. Red arrows refer to pathways investigated further. Figure S5. Heatmap referring to enriched enzymes of microbiomes of rhizosphere (R) and surrounding bulk (S) soils of A. fruticosum. Red arrows refer to the most enriched enzymes that were investigated further. Detailed information for the different enzymes are shown in Table S15. Figure S6. KEGG pathway “Quorum sensing” of sub-category “Cellular community - prokaryotes” (category “Cellular Processes”) referring to the enriched steps at varying levels in rhizospheric microbiome of A. fruticosum. Blue arrow refers to the step with the enriched enzyme, e.g., long-chain acyl-CoA synthetase (EC 6.2.1.3), in the pathway. Colored boxes around enzyme EC or metabolite refer to the enrichment level, where red refers to the high level compared with that of the bulk soil, while blue refers to the low level compared with that of the bulk soil. See scale in the figure for intermediate enrichment levels. Figure S7. KEGG pathway “ABC transporters” of sub-category “Membrane transport” (category “Environmental Information Processing”) referring to the enriched steps at varying levels in rhiz [file 13568_2024_1678_MOESM1_ESM.docx]

Figure S1. Gene number of functional categories and sub-categories from KEGG database across rhizospheric and bulk soil microbiomes of *A. fruticosum*. In this figure, we present a comprehensive depiction of the gene number distribution pertaining to diverse functional categories and sub-categories derived from the KEGG database. These data have been meticulously collected and analyzed across both the rhizospheric microbiome and the surrounding bulk soil ecosystem associated with *A. fruticosum*. Of notable significance, we employ red arrows to demarcate those sub-categories that have been subjected to in-depth investigation, thereby serving as critical foci for further examination and elucidation.

Figure S2. Gene abundance heatmap illustrating KEGG categories in rhizospheric and surrounding bulk soil microbiomes of *A. fruticosum*. This figure showcases an intricately detailed heatmap representation, offering insights into the distribution of gene abundances across the diverse KEGG categories within the microbiomes of both the rhizosphere and adjacent bulk soil habitat surrounding *A. fruticosum*. The discerning observer will identify the deployment of red arrows to emphasize specific categories that have been subjected to a more intensive analytical scrutiny, thereby directing scholarly attention toward the most pertinent areas of investigation.

Figure S3. Gene abundance heatmap visualizing KEGG sub-categories in *A. fruticosum*'s rhizospheric and surrounding bulk soil microbiomes. This figure artfully presents a heatmap, wherein the distribution of gene abundances related to KEGG sub-categories within the rhizospheric and surrounding bulk soil microbiomes of *A. fruticosum* is artistically conveyed. The judicious application of red arrows conspicuously highlights particular sub-categories of utmost scientific intrigue, thereby guiding rigorous inquiry and exploration.

Figure S4. Gene abundance heatmap representing KEGG pathways in *A. fruticosum*'s rhizospheric and surrounding bulk soil microbiomes. Within this figure, we offer a meticulously crafted heatmap that unveils the intricate patterns of gene abundance across various KEGG pathways present within the rhizospheric and adjacent bulk soil microbiomes affiliated with *A. fruticosum*. The strategic employment of red arrows efficiently directs the reader's attention towards specific pathways that have been subjected to meticulous investigation, thereby facilitating focused and incisive scientific inquiry.


Figure S5. Gene abundance heatmap visualizing ECs of enriched enzymes of *A. fruticosum*'s rhizospheric and surrounding bulk soil microbiomes. In this figure, we present an enriching heatmap that elegantly elucidates the distribution of enriched enzymes across the microbiomes of both the rhizosphere and the surrounding bulk soil linked to *A. fruticosum*. To navigate through this intricate landscape, one can rely on the red arrows, strategically deployed to highlight the most enriched enzymes that have undergone a comprehensive and rigorous investigation. Further insights into the specifics of these enzymes are elegantly expounded in Table S16.

Figure S6. KEGG pathway “Quorum sensing” of sub-category “Cellular community - prokaryotes” (category “Cellular Processes”) referring to the enriched steps at varying levels in rhizospheric microbiome of *A. fruticosum*. Blue arrow refers to the step with the enriched enzyme, e.g., long-chain acyl-CoA synthetase (EC 6.2.1.3), in the pathway. The discernment of differential enrichment levels is facilitated by the judicious application of colored boxes surrounding the respective enzyme EC or metabolite, wherein the red conveys an elevated enrichment level in comparison to the bulk soil counterpart. Correspondingly, the blue box signifies a relatively reduced enrichment level vis-à-vis the bulk soil. Notably, for a comprehensive grasp of intermediary enrichment gradations, the figure proffers a dedicated scale.


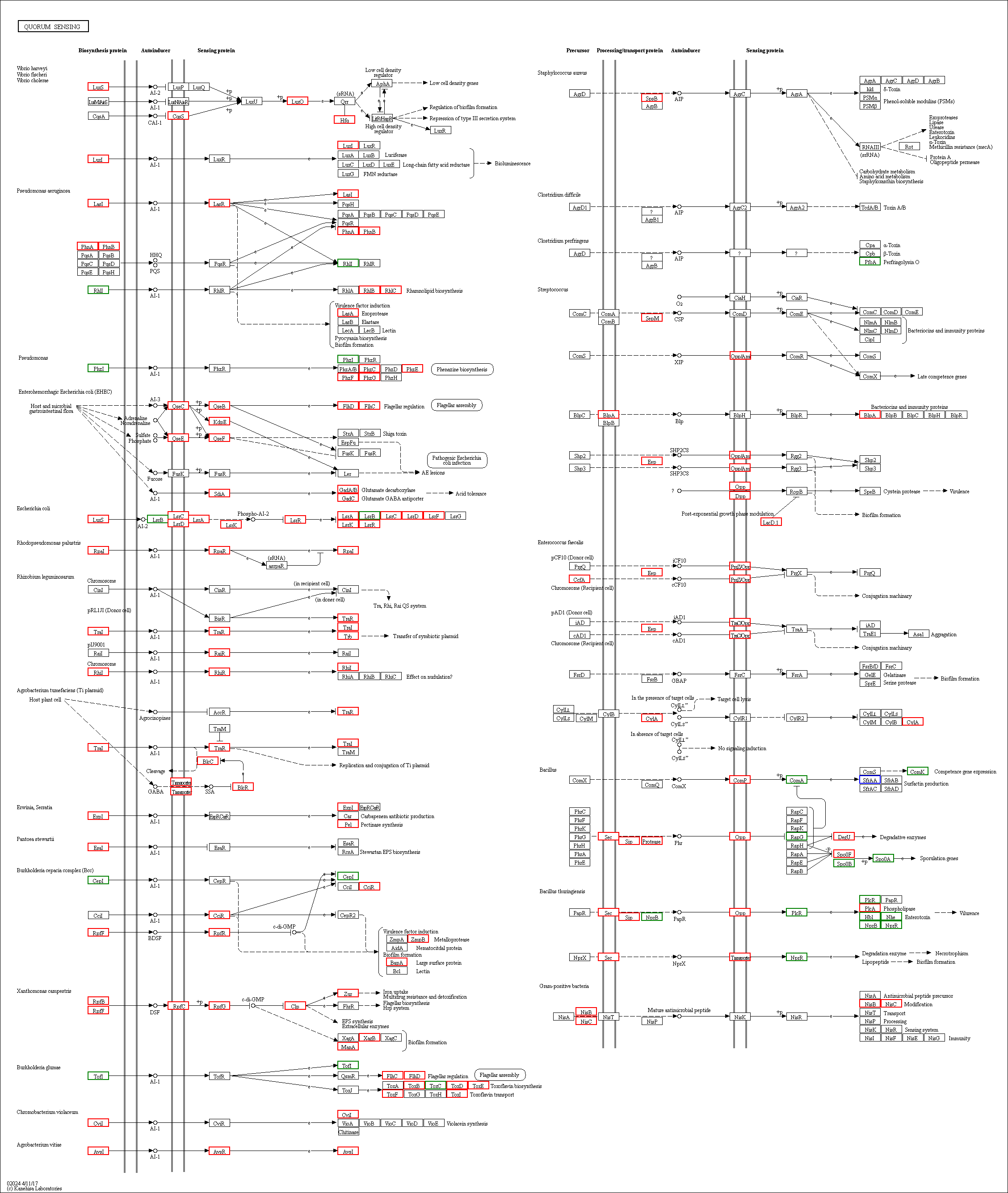

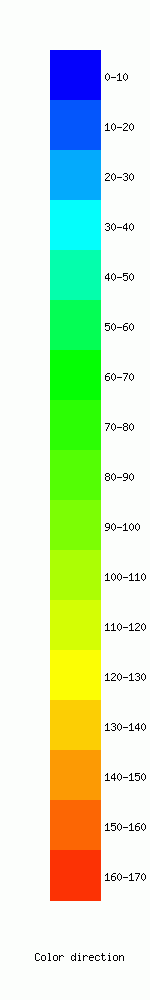


Figure S7. KEGG pathway “ABC transporters” of sub-category “Membrane transport” (category “Environmental Information Processing”) referring to the enriched steps at varying levels in rhizospheric microbiome of *A. fruticosum*. Blue arrow refers to the step with the most enriched enzyme, e.g., ribose transport system ATP-binding protein (EC 3.6.3.17/7.5.2.7), in the pathway. The discernment of differential enrichment levels is facilitated by the judicious application of colored boxes surrounding the respective enzyme EC or metabolite, wherein the red conveys an elevated enrichment level in comparison to the bulk soil counterpart. Correspondingly, the blue box signifies a relatively reduced enrichment level vis-à-vis the bulk soil. Notably, for a comprehensive grasp of intermediary enrichment gradations, the figure proffers a dedicated scale.


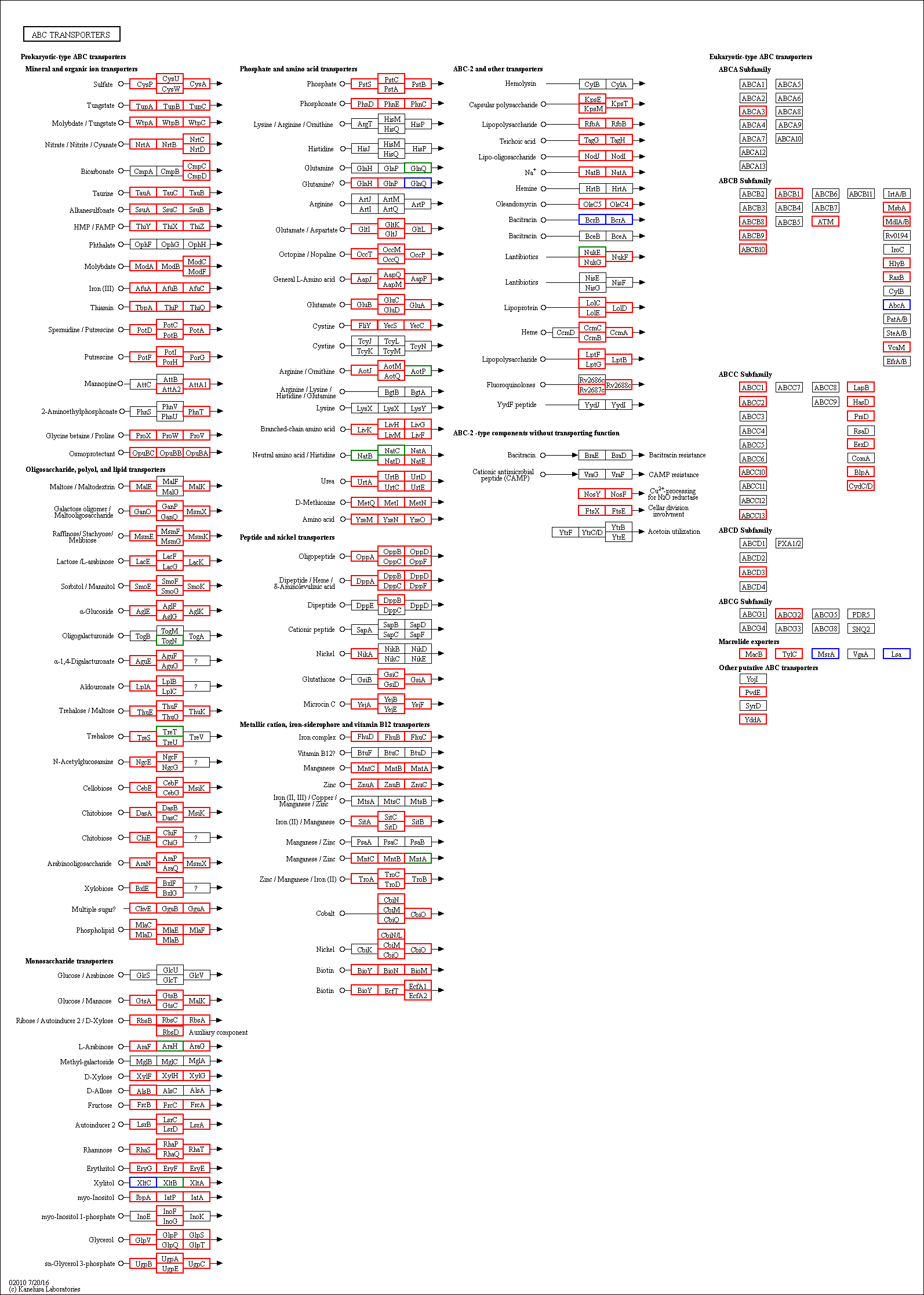

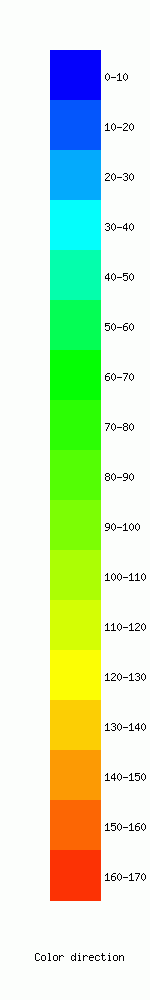


Figure S8. KEGG pathway “Two-component system” of sub-category “Signal transduction” (category “Environmental Information Processing”) referring to the enriched steps at varying levels in rhizospheric microbiome of *A. fruticosum*. Blue arrow refers to the step with the most enriched enzyme, e.g., phosphate regulon sensor histidine kinase PhoR (EC 2.7.13.3), in the pathway. The discernment of differential enrichment levels is facilitated by the judicious application of colored boxes surrounding the respective enzyme EC or metabolite, wherein the red conveys an elevated enrichment level in comparison to the bulk soil counterpart. Correspondingly, the blue box signifies a relatively reduced enrichment level vis-à-vis the bulk soil. Notably, for a comprehensive grasp of intermediary enrichment gradations, the figure proffers a dedicated scale.


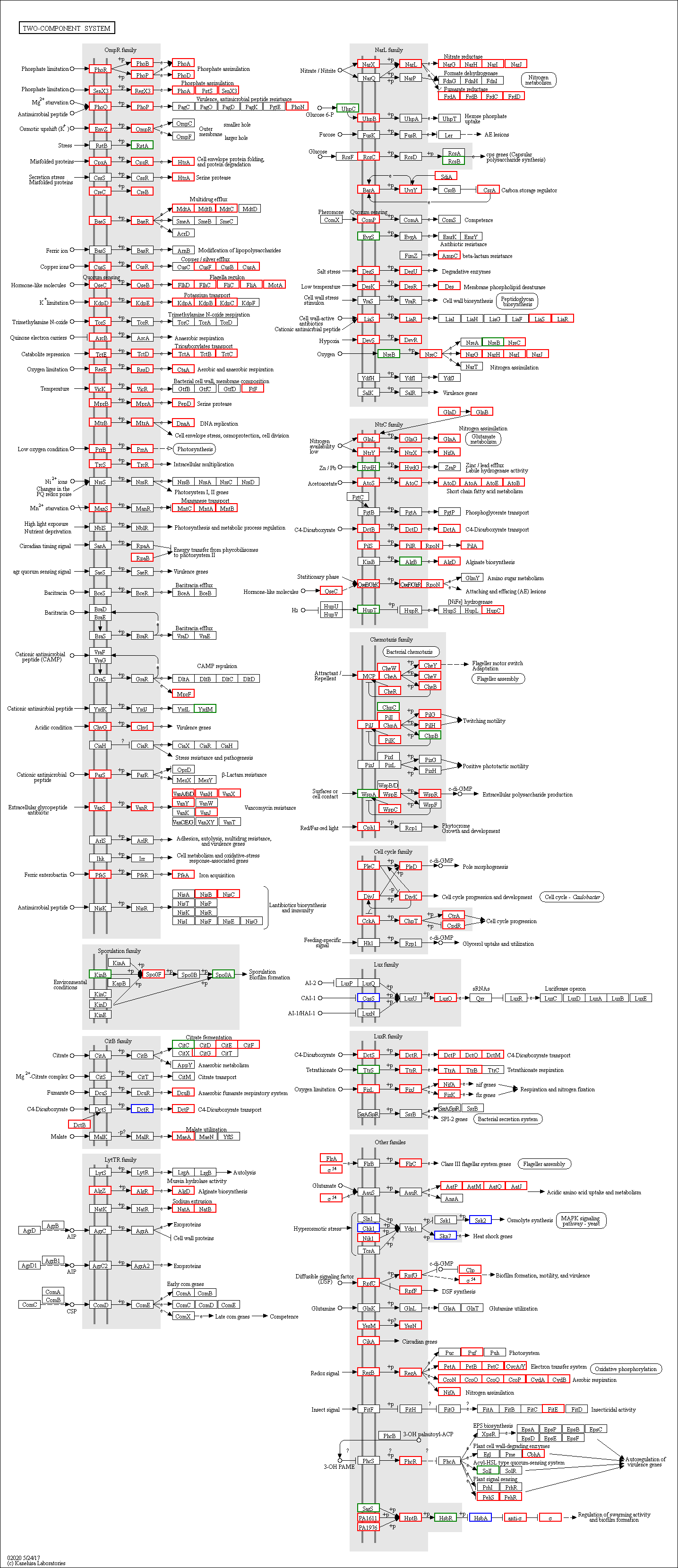

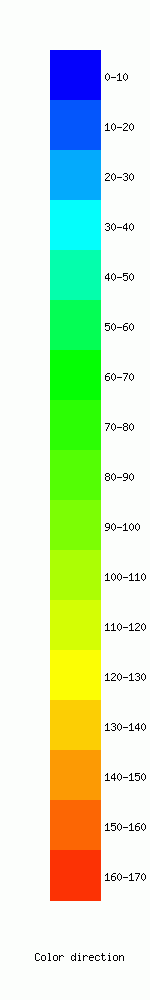


Figure S9. KEGG pathway “Aminoacyl-tRNA biosynthesis” of sub-category “Translation” (category “Genetic Information Processing”) referring to ECs of the enriched enzymes at varying levels in rhizospheric microbiome of *A. fruticosum*. Blue arrow refers to the step with the most enriched enzyme, e.g., aspartyl-tRNA(Asn)/glutamyl-tRNA(Gln) amidotransferase subunit B (EC 6.3.5.6/6.3.5.7), in the pathway. The discernment of differential enrichment levels is facilitated by the judicious application of colored boxes surrounding the respective enzyme EC or metabolite, wherein the red conveys an elevated enrichment level in comparison to the bulk soil counterpart. Correspondingly, the blue box signifies a relatively reduced enrichment level vis-à-vis the bulk soil. Notably, for a comprehensive grasp of intermediary enrichment gradations, the figure proffers a dedicated scale.


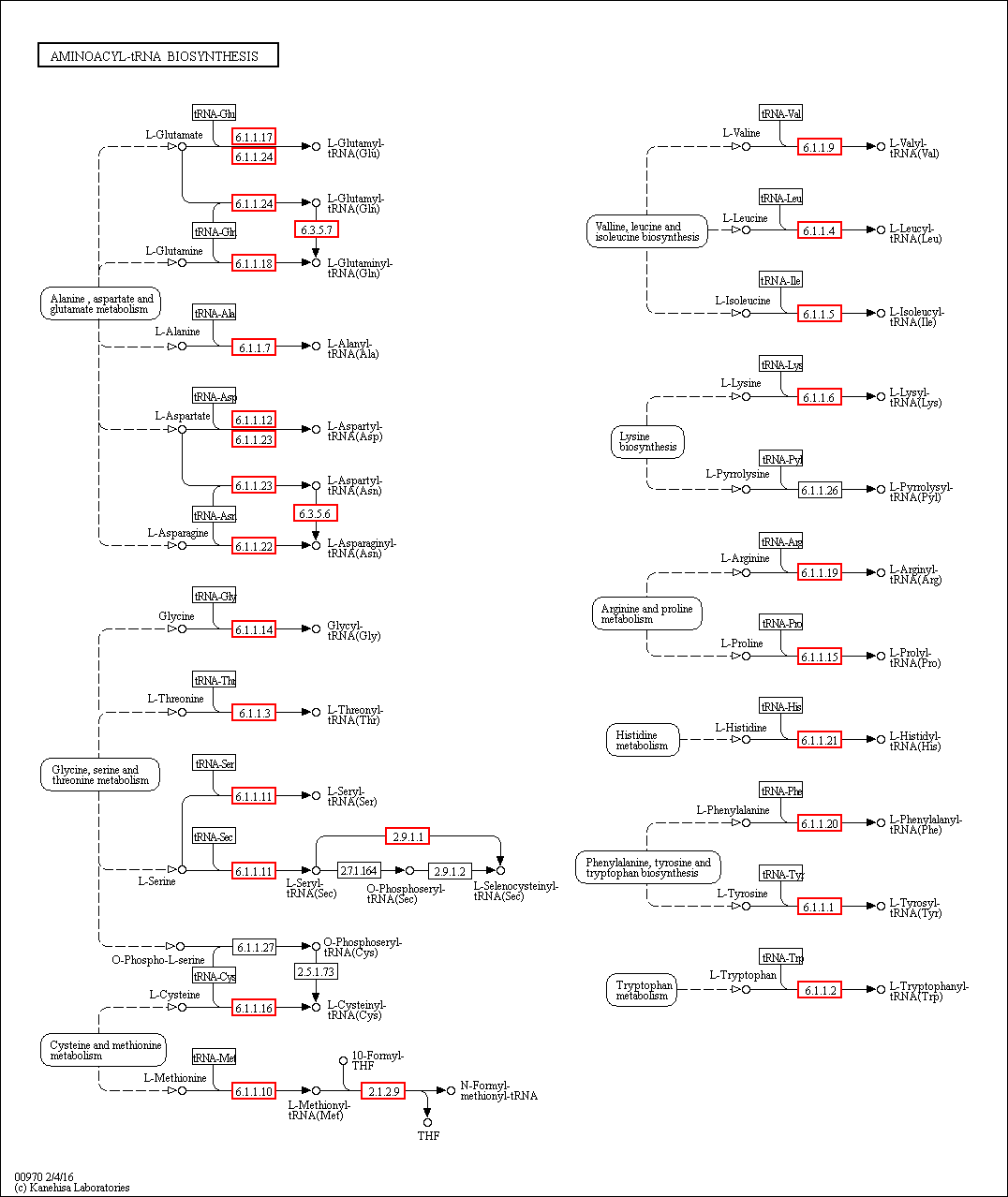

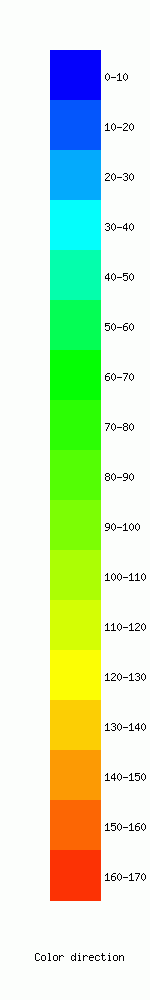


Figure S10. KEGG pathway “Valine, leucine and isoleucine biosynthesis” of sub-category “Amino acids metabolism” (category “Metabolism”) referring to ECs of the enriched enzymes at varying levels in rhizospheric microbiome of *A. fruticosum*. Blue arrow refers to the step with the most enriched enzyme, e.g., acetolactate synthase I/II/III large subunit (EC 2.2.1.6), in the pathway. The discernment of differential enrichment levels is facilitated by the judicious application of colored boxes surrounding the respective enzyme EC or metabolite, wherein the red conveys an elevated enrichment level in comparison to the bulk soil counterpart. Correspondingly, the blue box signifies a relatively reduced enrichment level vis-à-vis the bulk soil. Notably, for a comprehensive grasp of intermediary enrichment gradations, the figure proffers a dedicated scale.


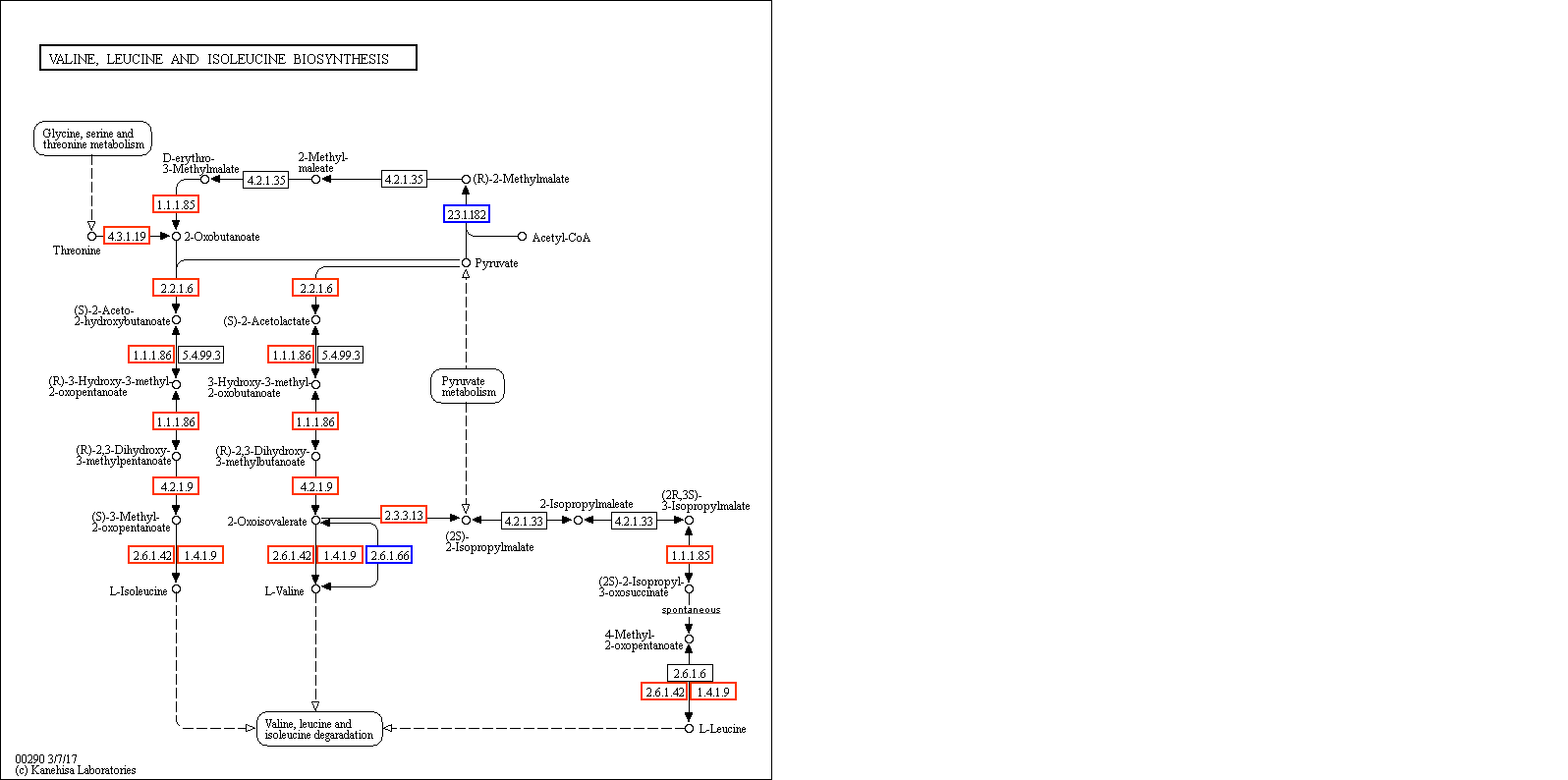

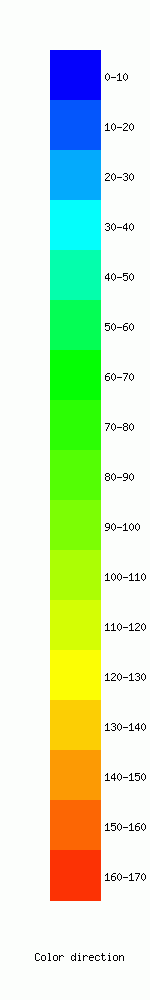


Figure S11. KEGG pathway “Valine, leucine and isoleucine degradation” of sub-category “Amino acids metabolism” (category “Metabolism”) referring to ECs of the enriched enzymes at varying levels in rhizospheric microbiome of *A. fruticosum*. Blue arrow refers to the step with the most enriched enzyme, e.g., acetyl-CoA C-acetyltransferase (EC 2.3.1.9), in the pathway. The discernment of differential enrichment levels is facilitated by the judicious application of colored boxes surrounding the respective enzyme EC or metabolite, wherein the red conveys an elevated enrichment level in comparison to the bulk soil counterpart. Correspondingly, the blue box signifies a relatively reduced enrichment level vis-à-vis the bulk soil. Notably, for a comprehensive grasp of intermediary enrichment gradations, the figure proffers a dedicated scale.


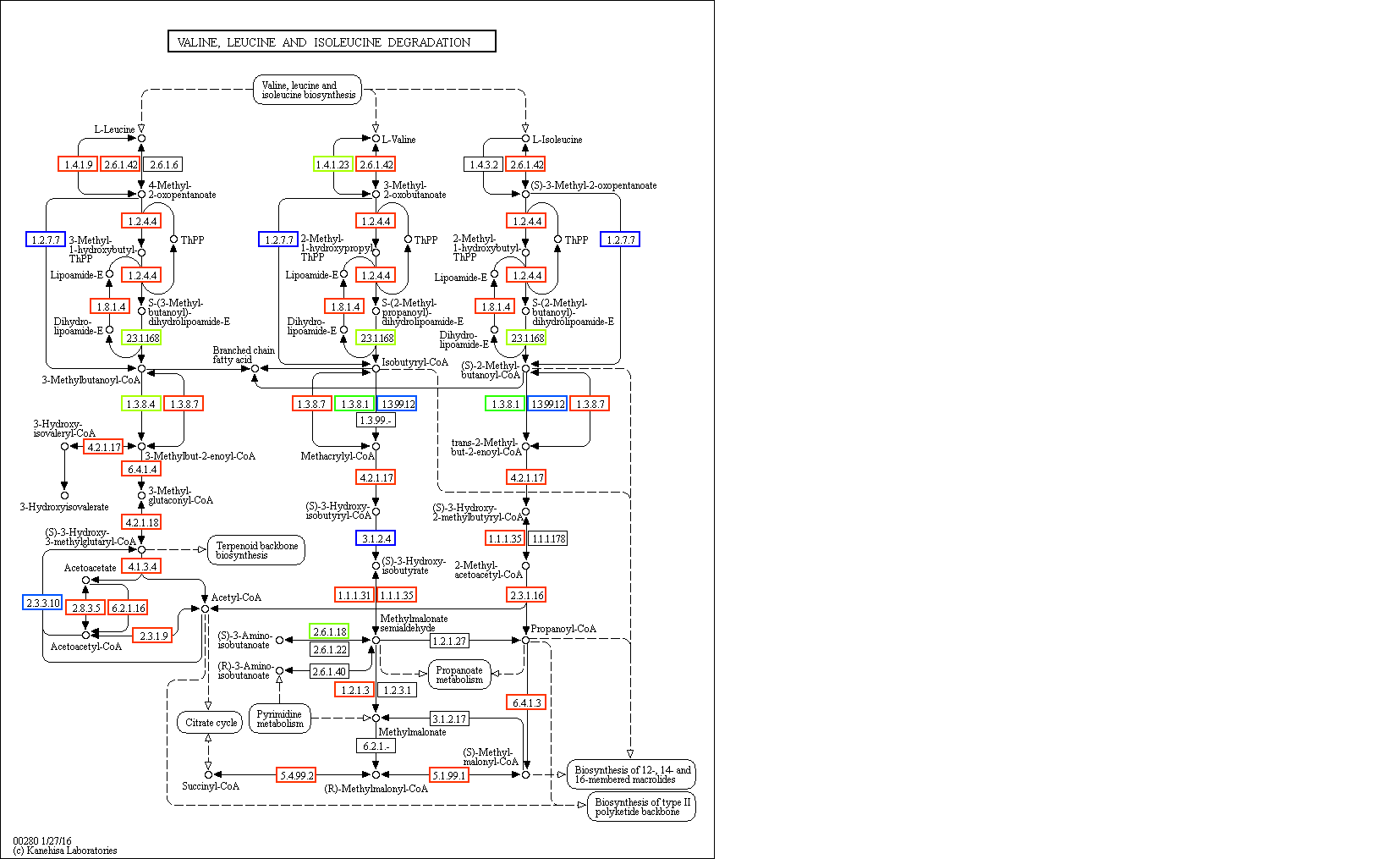

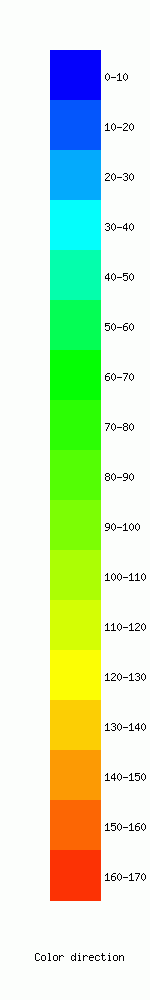


Figure S12. KEGG pathway “Glycolysis / Gluconeogenesis” of sub-category “Carbohydrate metabolism” (category “Metabolism”) referring to ECs of the enriched enzymes at varying levels in rhizospheric microbiome of *A. fruticosum*. Blue arrow refers to the step with the most enriched enzyme, e.g., pyruvate dehydrogenase E1 component beta subunit (EC 1.2.4.1), in the pathway. The discernment of differential enrichment levels is facilitated by the judicious application of colored boxes surrounding the respective enzyme EC or metabolite, wherein the red conveys an elevated enrichment level in comparison to the bulk soil counterpart. Correspondingly, the blue box signifies a relatively reduced enrichment level vis-à-vis the bulk soil. Notably, for a comprehensive grasp of intermediary enrichment gradations, the figure proffers a dedicated scale.


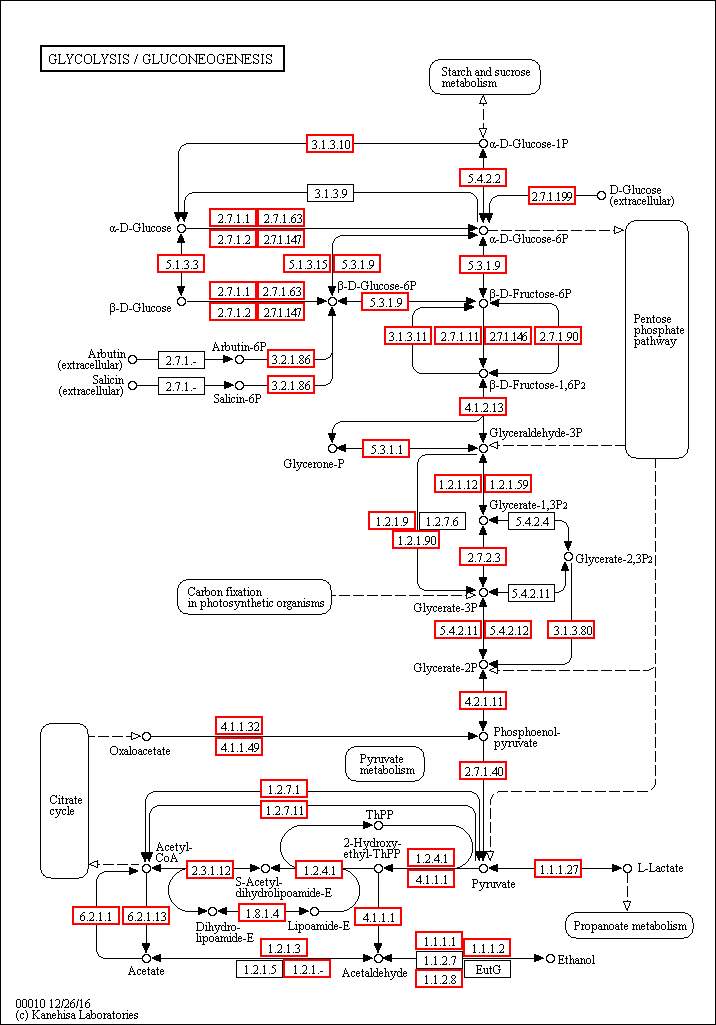

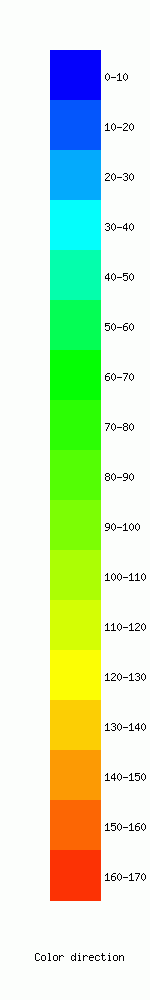


Figure S13. KEGG pathway “Citrate cycle (TCA cycle)” of sub-category “Carbohydrate metabolism” (category “Metabolism”) referring to ECs of the enriched enzymes at varying levels in rhizospheric microbiome of *A. fruticosum*. Blue arrow refers to the step with the most enriched enzyme, e.g., pyruvate dehydrogenase E1 component beta subunit (EC 1.2.4.1), in the pathway. The discernment of differential enrichment levels is facilitated by the judicious application of colored boxes surrounding the respective enzyme EC or metabolite, wherein the red conveys an elevated enrichment level in comparison to the bulk soil counterpart. Correspondingly, the blue box signifies a relatively reduced enrichment level vis-à-vis the bulk soil. Notably, for a comprehensive grasp of intermediary enrichment gradations, the figure proffers a dedicated scale.


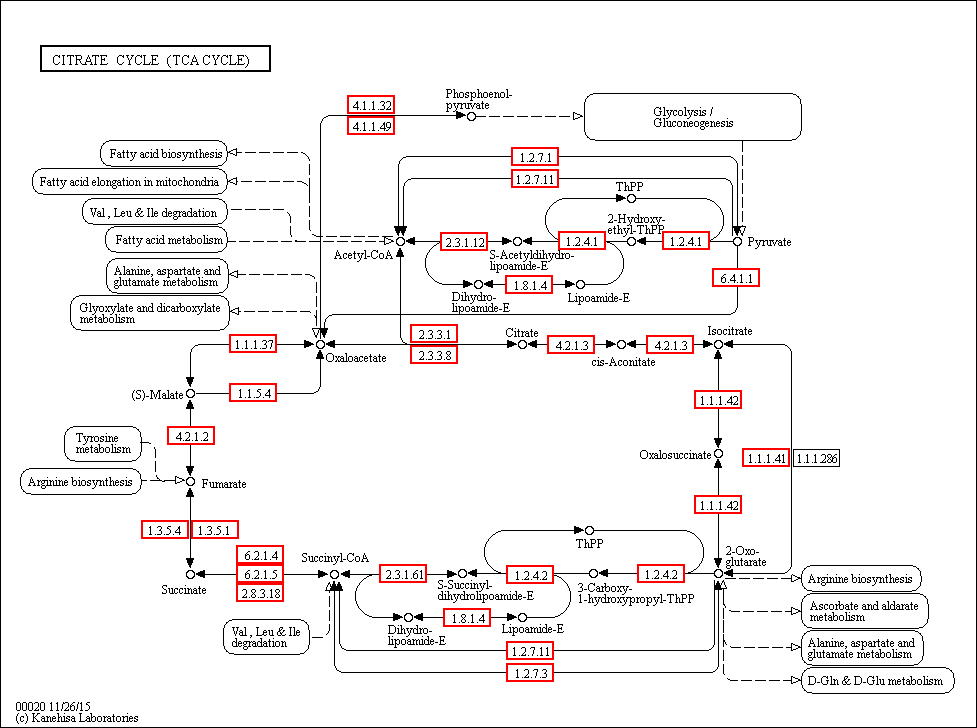

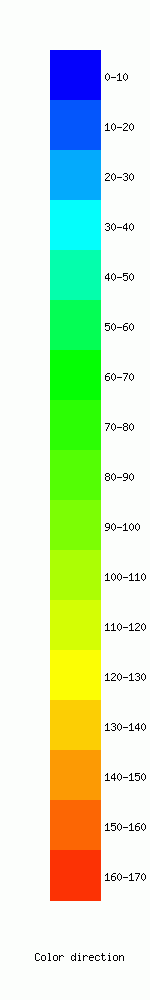


Figure S14. KEGG pathway “Pyruvate metabolism” of sub-category “Carbohydrate metabolism” (category “Metabolism”) referring to ECs of the enriched enzymes at varying levels in rhizospheric microbiome of *A. fruticosum*. Blue arrows refer to the steps with the most enriched enzymes, e.g., acetyl-CoA C-acetyltransferase (EC 2.3.1.9) and pyruvate dehydrogenase E1 component beta subunit (EC 1.2.4.1), in the pathway. The discernment of differential enrichment levels is facilitated by the judicious application of colored boxes surrounding the respective enzyme EC or metabolite, wherein the red conveys an elevated enrichment level in comparison to the bulk soil counterpart. Correspondingly, the blue box signifies a relatively reduced enrichment level vis-à-vis the bulk soil. Notably, for a comprehensive grasp of intermediary enrichment gradations, the figure proffers a dedicated scale.


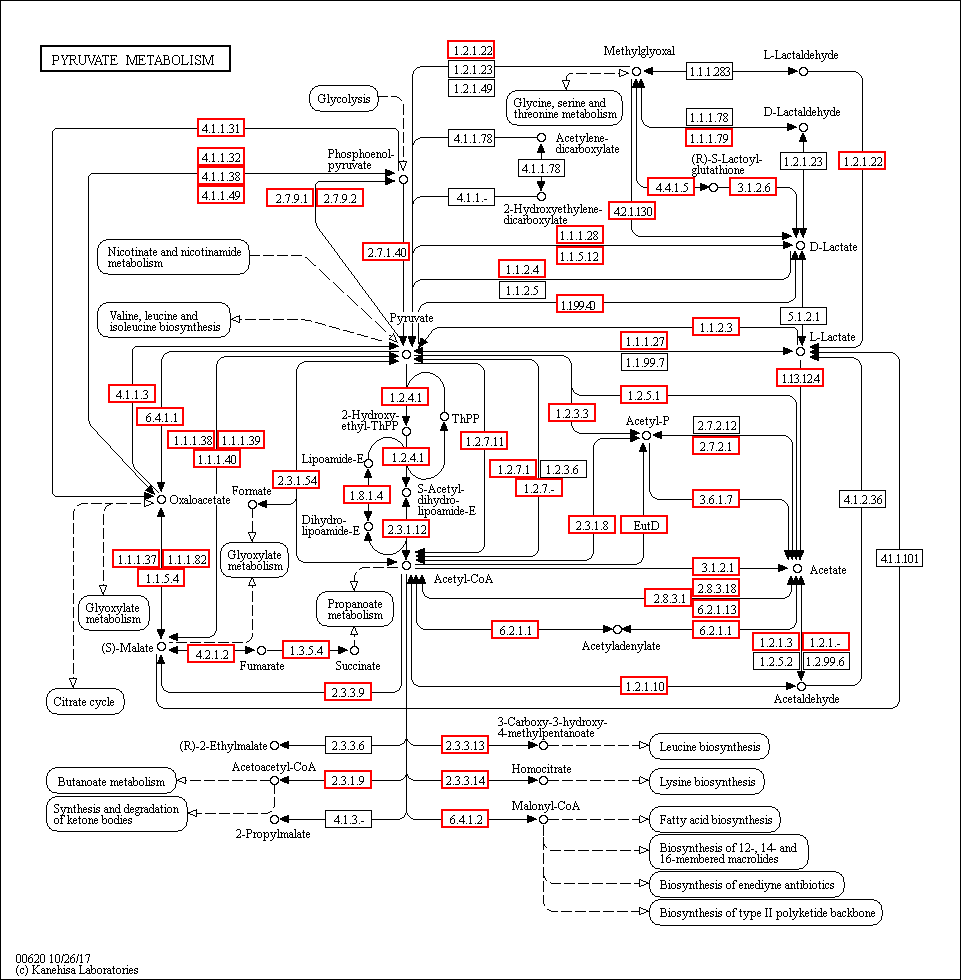

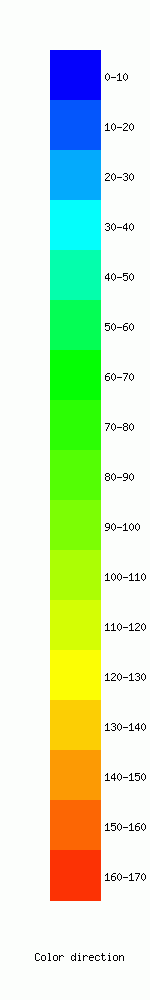


Figure S15. KEGG pathway “Glyoxylate and dicarboxylate metabolism” of sub-category “Carbohydrate metabolism” (category “Metabolism”) referring to ECs of the enriched enzymes at varying levels in rhizospheric microbiome of *A. fruticosum*. Blue arrow refers to the step with the most enriched enzyme, e.g., acetyl-CoA C-acetyltransferase (EC 2.3.1.9), in the pathway. The discernment of differential enrichment levels is facilitated by the judicious application of colored boxes surrounding the respective enzyme EC or metabolite, wherein the red conveys an elevated enrichment level in comparison to the bulk soil counterpart. Correspondingly, the blue box signifies a relatively reduced enrichment level vis-à-vis the bulk soil. Notably, for a comprehensive grasp of intermediary enrichment gradations, the figure proffers a dedicated scale.


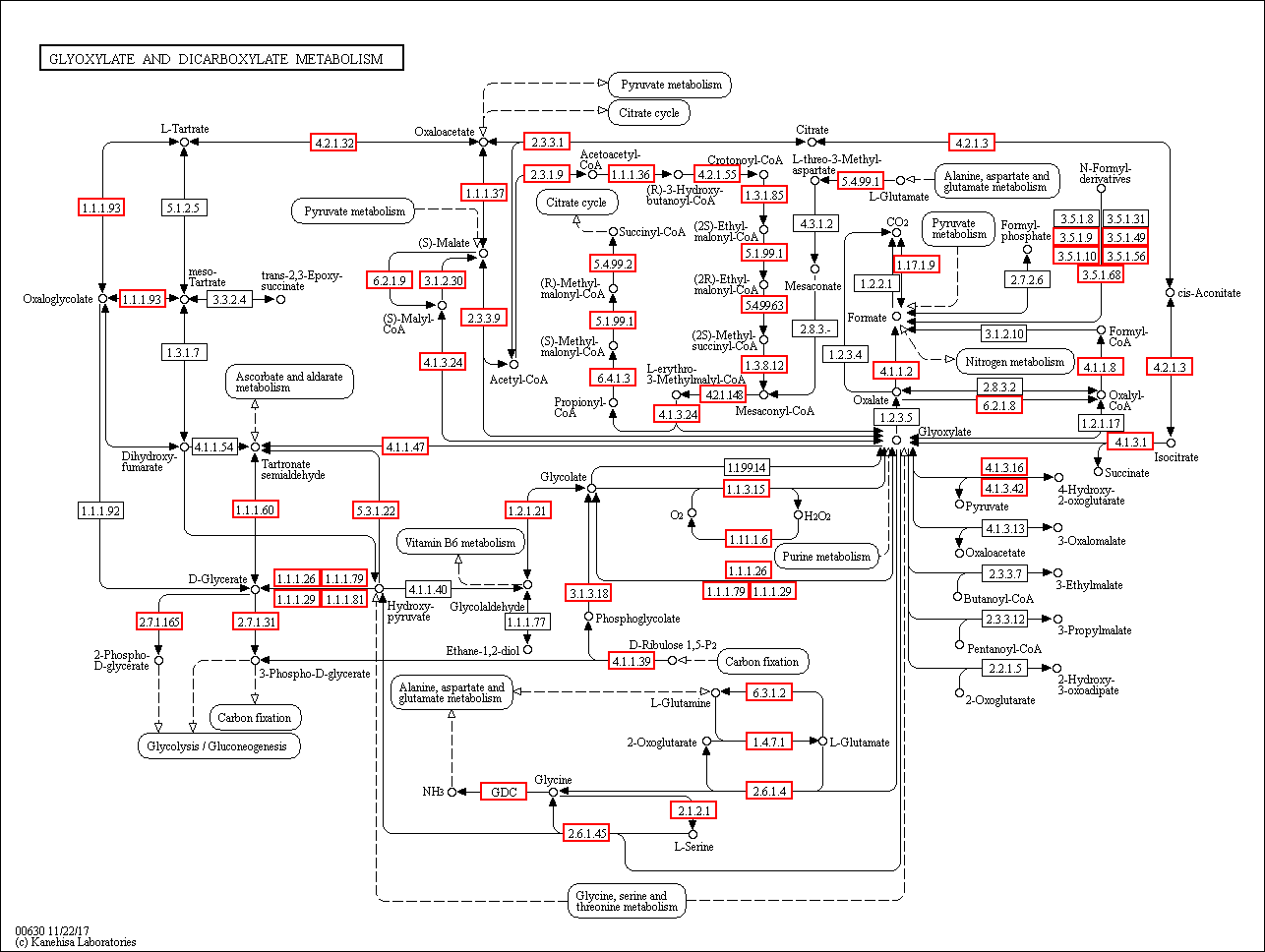

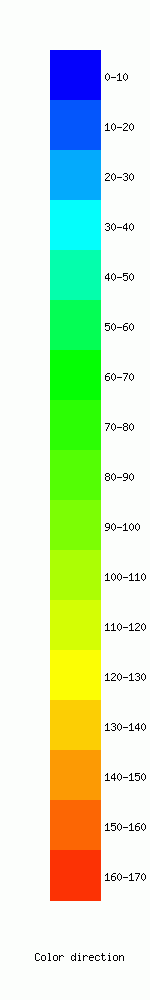


Figure S16. KEGG pathway “Oxidative phosphorylation” of sub-category “Energy metabolism” (category “Metabolism”) referring to ECs of the enriched enzymes at varying levels in rhizospheric microbiome of *A. fruticosum*. Blue arrows refer to the steps with the most enriched enzymes, e.g., NADH-quinone oxidoreductase subunit J (EC 1.6.5.3/7.1.1.2), cytochrome c oxidase subunit III (EC 1.9.3.1/7.1.1.9) and F-type H+-transporting ATPase subunit beta (EC 3.6.3.14/7.1.2.2), in the pathway. The discernment of differential enrichment levels is facilitated by the judicious application of colored boxes surrounding the respective enzyme EC or metabolite, wherein the red conveys an elevated enrichment level in comparison to the bulk soil counterpart. Correspondingly, the blue box signifies a relatively reduced enrichment level vis-à-vis the bulk soil. Notably, for a comprehensive grasp of intermediary enrichment gradations, the figure proffers a dedicated scale.


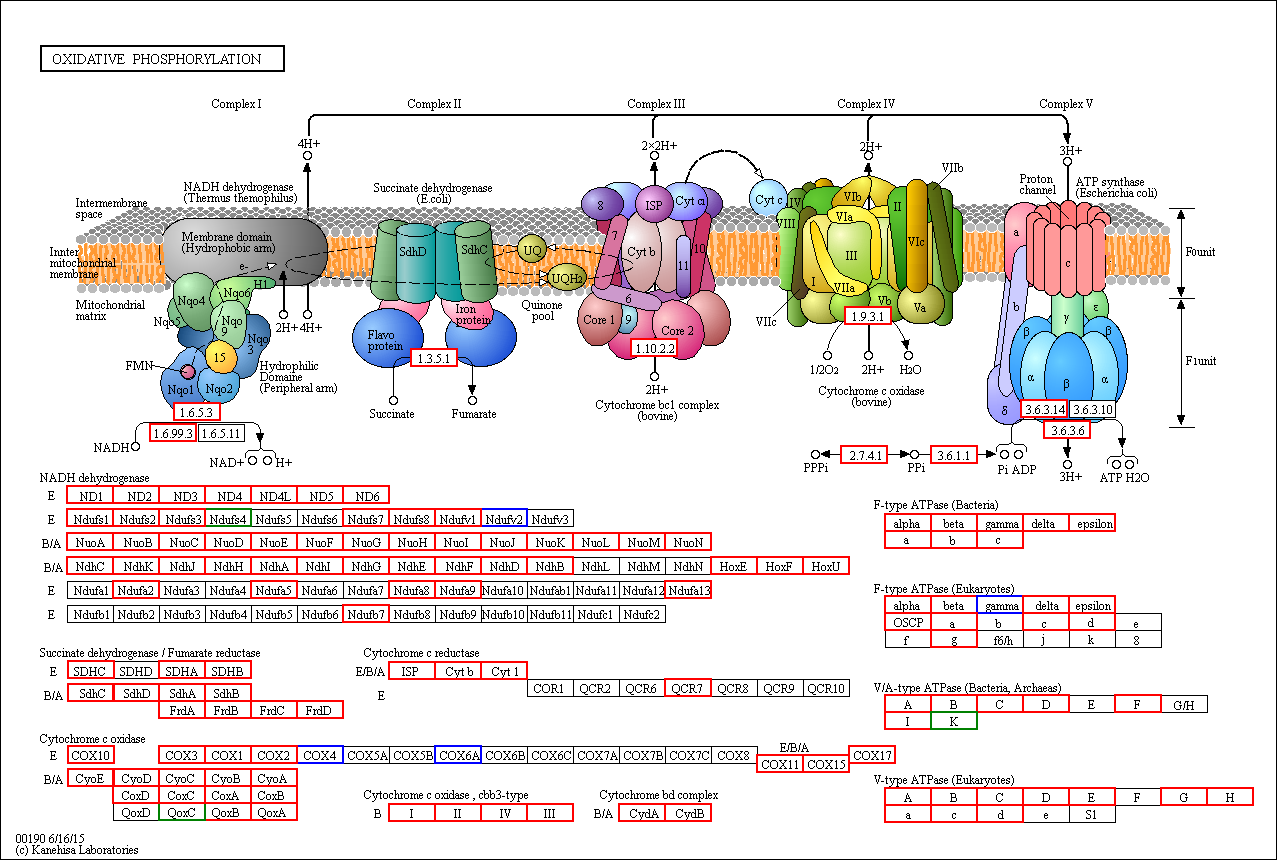

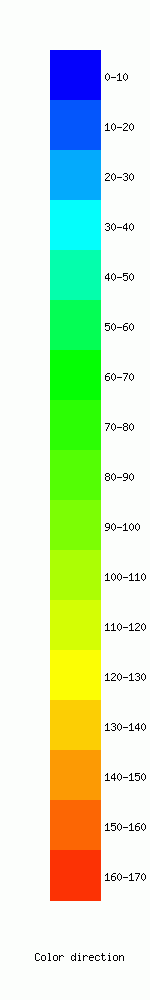


Figure S17. KEGG pathway “Carbon fixation pathways in prokaryotes” of sub-category “Energy metabolism” (category “Metabolism”) referring to ECs of the enriched enzymes at varying levels in rhizospheric microbiome of *A. fruticosum*. Blue arrow refers to the step with the most enriched enzyme, e.g., acetyl-CoA C-acetyltransferase (EC 2.3.1.9), in the pathway. The discernment of differential enrichment levels is facilitated by the judicious application of colored boxes surrounding the respective enzyme EC or metabolite, wherein the red conveys an elevated enrichment level in comparison to the bulk soil counterpart. Correspondingly, the blue box signifies a relatively reduced enrichment level vis-à-vis the bulk soil. Notably, for a comprehensive grasp of intermediary enrichment gradations, the figure proffers a dedicated scale.


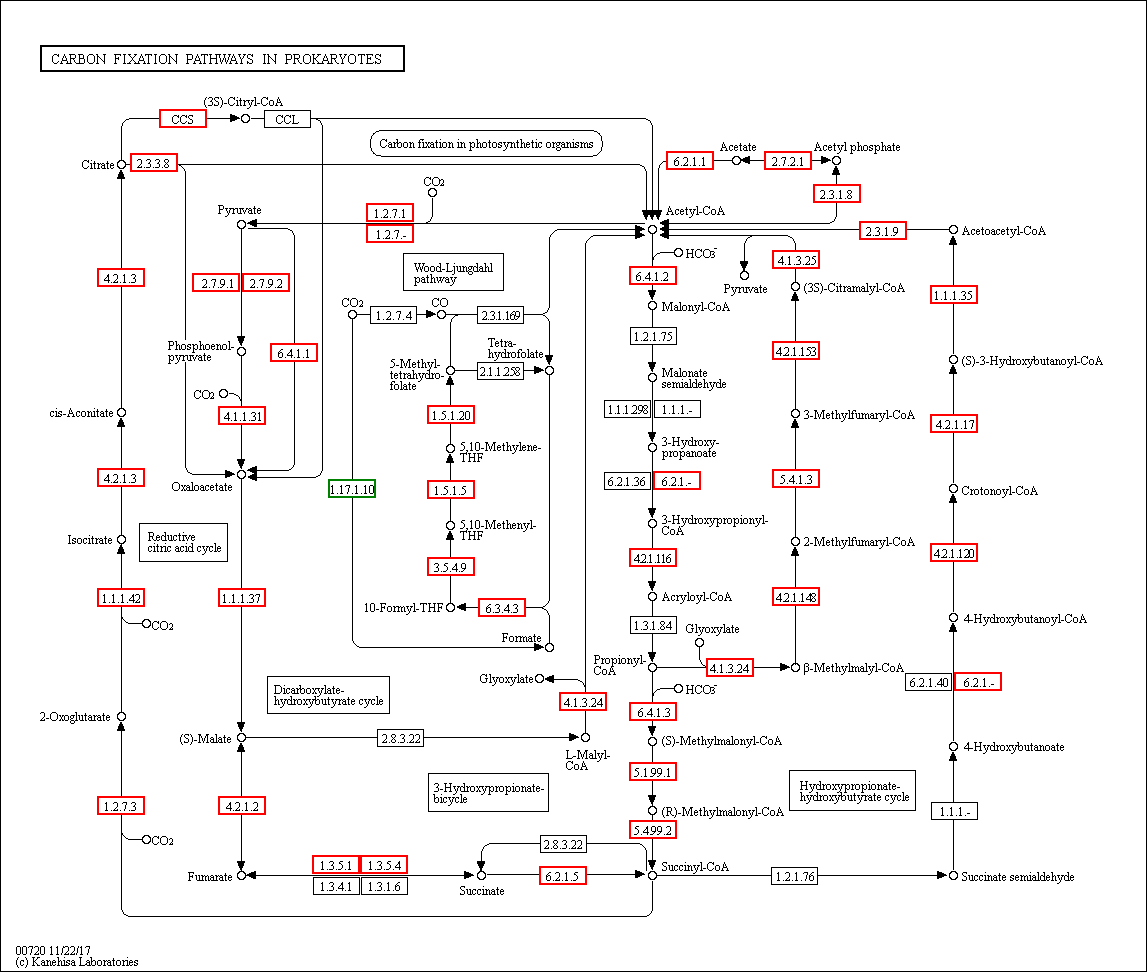

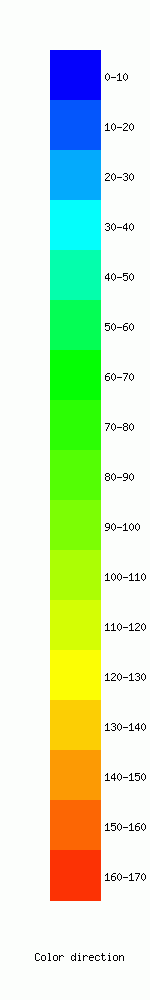


Figure S18. KEGG pathway “Purine metabolism” of sub-category “Nucleotide metabolism” (category “Metabolism”) referring to ECs of the enriched enzymes at varying levels in rhizospheric microbiome of *A. fruticosum*. Blue arrows refer to the steps with the most enriched enzymes, e.g., DNA-directed RNA polymerase subunit beta' (EC 2.7.7.6), DNA polymerase III subunit delta (EC 2.7.7.7) and nucleoside-diphosphate kinase (EC 2.7.4.6), in the pathway. The discernment of differential enrichment levels is facilitated by the judicious application of colored boxes surrounding the respective enzyme EC or metabolite, wherein the red conveys an elevated enrichment level in comparison to the bulk soil counterpart. Correspondingly, the blue box signifies a relatively reduced enrichment level vis-à-vis the bulk soil. Notably, for a comprehensive grasp of intermediary enrichment gradations, the figure proffers a dedicated scale.


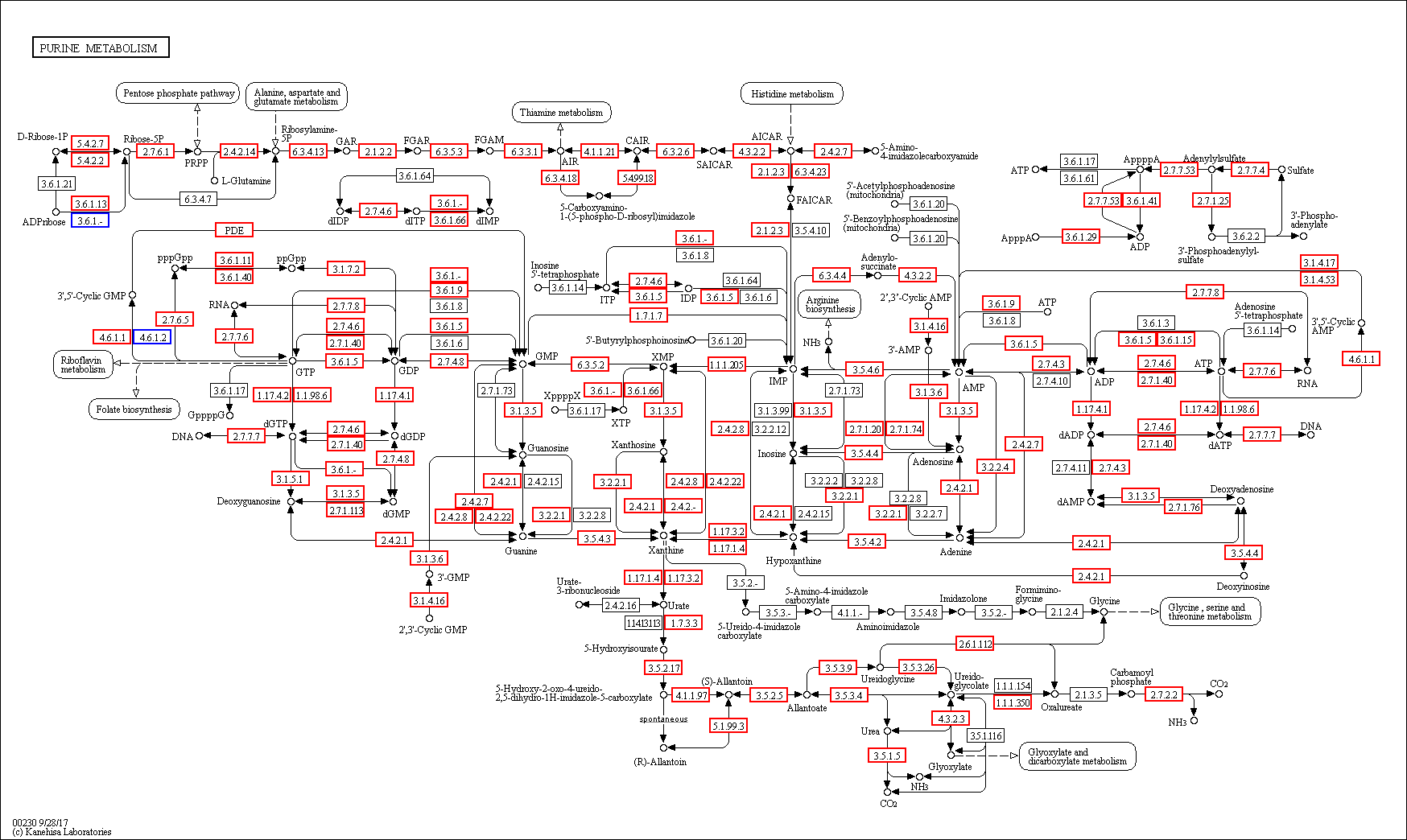

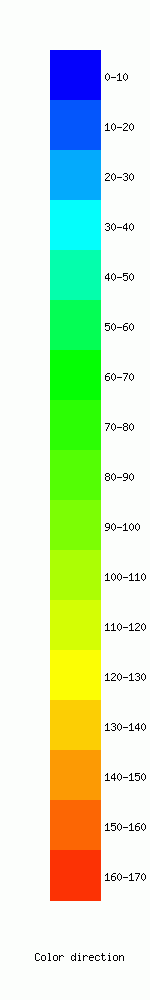


Figure S19. KEGG pathway “Pyrimidine metabolism” of sub-category “Nucleotide metabolism” (category “Metabolism”) referring to ECs of the enriched enzymes at varying levels in rhizospheric microbiome of *A. fruticosum*. Blue arrows refer to the steps with the most enriched enzymes, e.g., DNA-directed RNA polymerase subunit beta' (EC 2.7.7.6), DNA polymerase III subunit delta (EC 2.7.7.7), nucleoside-diphosphate kinase (EC 2.7.4.6), carbamoyl-phosphate synthase large subunit (EC 6.3.5.5) and CMP/dCMP kinase (EC 2.7.4.25), in the pathway. The discernment of differential enrichment levels is facilitated by the judicious application of colored boxes surrounding the respective enzyme EC or metabolite, wherein the red conveys an elevated enrichment level in comparison to the bulk soil counterpart. Correspondingly, the blue box signifies a relatively reduced enrichment level vis-à-vis the bulk soil. Notably, for a comprehensive grasp of intermediary enrichment gradations, the figure proffers a dedicated scale.


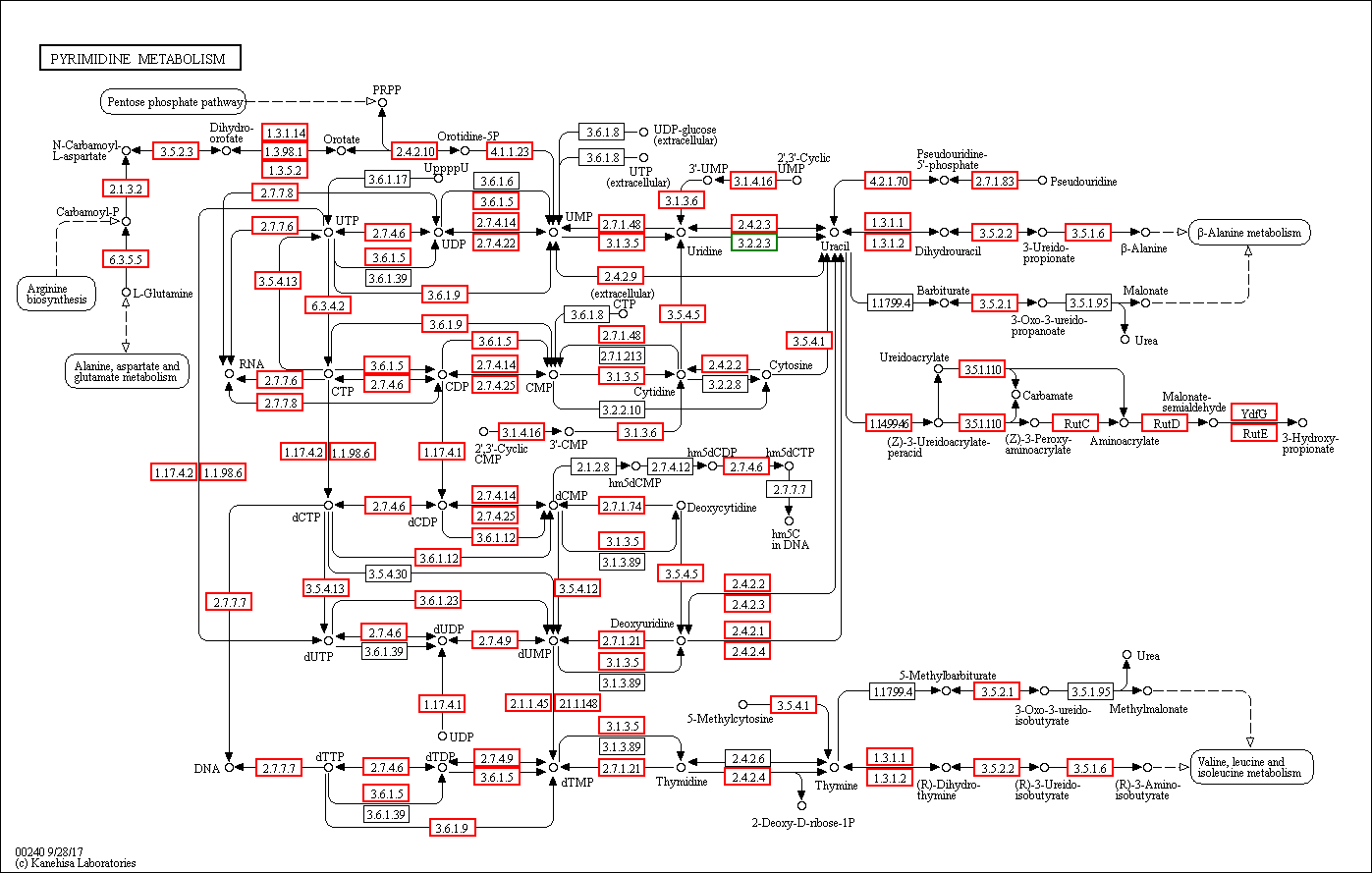

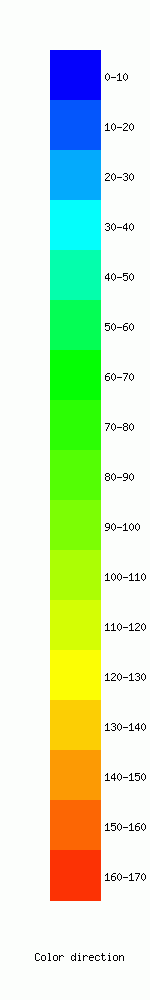


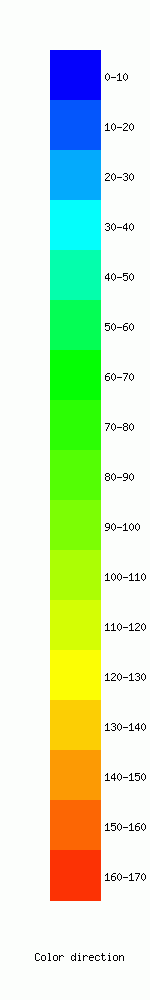

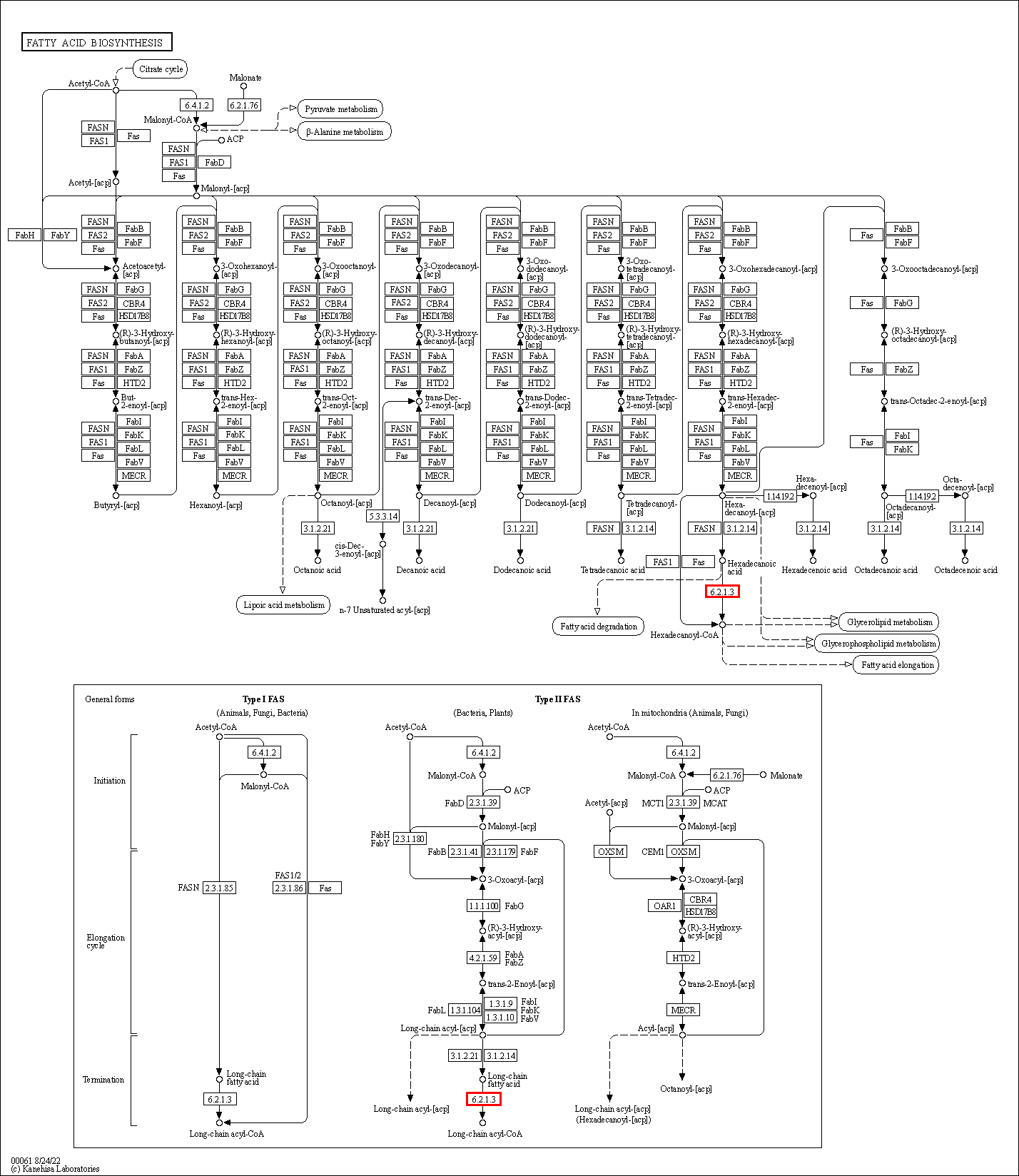
Figure S20. The less enriched KEGG pathway “Fatty acid biosynthesis” of sub-category “Lipid metabolism” (category “Metabolism”) that refers to the highly enriched enzyme, e.g., long-chain acyl-CoA synthetase (EC 6.2.1.3), in the pathway. The discernment of differential enrichment levels is facilitated by the judicious application of colored boxes surrounding the respective enzyme EC or metabolite, wherein the red conveys an elevated enrichment level in comparison to the bulk soil counterpart. Correspondingly, the blue box signifies a relatively reduced enrichment level vis-à-vis the bulk soil. Notably, for a comprehensive grasp of intermediary enrichment gradations, the figure proffers a dedicated scale.
